# Low Carbon Footprint Recycling of Post‐Consumer PET Plastic with a Metagenomic Polyester Hydrolase

**DOI:** 10.1002/cssc.202101062

**Published:** 2022-02-10

**Authors:** Christian Sonnendecker, Juliane Oeser, P. Konstantin Richter, Patrick Hille, Ziyue Zhao, Cornelius Fischer, Holger Lippold, Paula Blázquez‐Sánchez, Felipe Engelberger, César A. Ramírez‐Sarmiento, Thorsten Oeser, Yuliia Lihanova, Ronny Frank, Heinz‐Georg Jahnke, Susan Billig, Bernd Abel, Norbert Sträter, Jörg Matysik, Wolfgang Zimmermann

**Affiliations:** ^1^ Institute of Analytical Chemistry Leipzig University 04103 Leipzig Germany; ^2^ Department of Microbiology and Bioprocess Technology Institute of Biochemistry Leipzig University 04103 Leipzig Germany; ^3^ Institute of Bioanalytical Chemistry, Centre for Biotechnology and Biomedicine Leipzig University 04103 Leipzig Germany; ^4^ Helmholtz-Zentrum Dresden-Rossendorf (HZDR) Institut für Ressourcenökologie Abteilung Reaktiver Transport D-04318 Leipzig Germany; ^5^ Institute for Biological and Medical Engineering Schools of Engineering, Medicine and Biological Sciences Pontificia Universidad Católica de Chile Santiago 7820436 Chile; ^6^ ANID—Millennium Science Initiative Program Millennium Institute for Integrative Biology (iBio) Santiago 8331150 Chile; ^7^ Centre for Biotechnology and Biomedicine Molecular Biological-Biochemical Processing Technology Leipzig University 04103 Leipzig Germany; ^8^ Leibniz Institute of Surface Engineering (IOM) Wilhelm-Ostwald-Institute of Physical and Theoretical Chemistry 04103 Leipzig Germany

**Keywords:** biocatalysis, hydrolases, metagenome, polymer degradation, recycling

## Abstract

Earth is flooded with plastics and the need for sustainable recycling strategies for polymers has become increasingly urgent. Enzyme‐based hydrolysis of post‐consumer plastic is an emerging strategy for closed‐loop recycling of polyethylene terephthalate (PET). The polyester hydrolase PHL7, isolated from a compost metagenome, completely hydrolyzes amorphous PET films, releasing 91 mg of terephthalic acid per hour and mg of enzyme. Vertical scanning interferometry shows degradation rates of the PET film of 6.8 μm h^−1^. Structural analysis indicates the importance of leucine at position 210 for the extraordinarily high PET‐hydrolyzing activity of PHL7. Within 24 h, 0.6 mg_enzyme_ g_PET_^−1^ completely degrades post‐consumer thermoform PET packaging in an aqueous buffer at 70 °C without any energy‐intensive pretreatments. Terephthalic acid recovered from the enzymatic hydrolysate is then used to synthesize virgin PET, demonstrating the potential of polyester hydrolases as catalysts in sustainable PET recycling processes with a low carbon footprint.

## Graphical abstract

**Tread lightly**: A highly efficient polyester hydrolase derived from plant compost metagenomes rapidly degrades amorphous polyethylene terephthalate (PET) at 70 °C. The direct hydrolysis of thermoform PET packaging flakes and the conversion of the recovered terephthalic acid into virgin PET demonstrate the potential of biocatalysis in plastic recycling processes with a low carbon footprint.

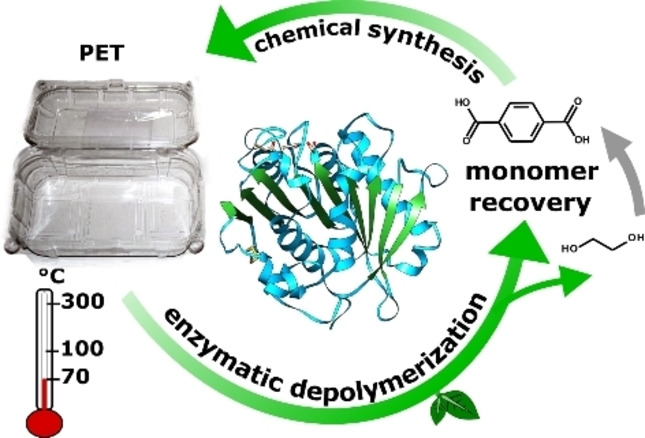

## Introduction

Plastics are ubiquitous and appear to be indispensable in our daily life. Due to their manifold applications and low cost of the petrochemical building blocks, the amount of plastics has immensely increased over the past decades. The worldwide production of plastics, which started in the 1950s, has resulted to date in an estimated amount of 8.3 billion metric tons.^[1]^ Only a small fraction is recycled, whereas the largest share has ended up in landfills or in the environment. Since synthetic plastics are not readily biodegradable, they accumulate in nature with detrimental effects on the environment and eventually on human health.^[2]^

Polyethylene terephthalate (PET) is a thermoplastic polymer consisting of the ester‐linked monomers terephthalic acid (TPA) and ethylene glycol (EG). Depending on its processing, PET exists either as an amorphous or as a semi‐crystalline polymer.^[3]^ PET is widely used in the food packaging industry and for the manufacture of textile fibers.^[4]^

The application of enzymes for the selective conversion of PET into the monomers at mild reaction conditions is emerging as a novel strategy for plastic recycling and valorization processes.[^5^, ^6^, ^7^, ^8^] An enzymatic hydrolysis can also be of advantage for the processing of waste composed of different types of plastics which are difficult to recycle by conventional recycling methods.^[9]^

Several enzymes of bacterial or fungal origin able to modify or degrade PET have been described.[^8^, ^10^] Thermostable cutinases (EC 3.1.1.74) and their homologues have been shown to be the most efficient catalysts to hydrolyze the ester bonds in PET yielding TPA, mono‐ and bis(2‐hydroxyethyl) terephthalic acid (MHET and BHET) and EG.[^5^, ^6^] Cutinases, evolved to degrade the aliphatic polyester cutin in plants, show a low substrate specificity which explains their ability to use synthetic polyesters such as PET, polyethylene furanoate, polybutylene succinate and polycaprolactone as substrates.^[11]^ An efficient enzymatic hydrolysis of PET requires a reaction temperature of about 70 °C where the plastic becomes more viscous at its glass transition temperature, allowing the enzyme to gain a better access to the polymer chains due to their increased mobility.[^12^, ^13^, ^14^]

The thermophilic cutinases from *Humicola insolens* (HiC) and from a plant compost metagenome (LCC) have previously shown the highest PET hydrolysis rates at reaction temperatures between 65 °C and 70 °C.[^14^, ^15^, ^16^] Recently, a hyperthermostable polyesterase with a melting temperature above 100 °C from a hot spring water metagenome^[17]^ with similar activity and 94 % sequence identity to LCC has also been reported.^[18]^ Mesophilic polyester hydrolases, for example from *Ideonella sakaiensis* ^[19]^ showed only low activity against PET compared to enzymes derived from thermophilic actinomycetes or fungi.[^14^, ^20^, ^21^, ^22^]

However, the hydrolytic activity of all polyester hydrolases reported so far appeared to be limited to the amorphous fraction of PET.[^13^, ^14^, ^23^, ^24^] A direct enzymatic hydrolysis of PET of higher crystallinity could therefore not be achieved.[^16^, ^24^] Herein, we report a novel polyester hydrolase (PHL7), isolated from plant composts with a high efficiency to degrade amorphous PET films and post‐consumer PET thermoform packaging. We compare its performance with the previously reported polyester hydrolase LCC and demonstrate a fast conversion of PET clamshell containers into the monomers without any pretreatments. The released TPA was subsequently used to produce virgin PET in a closed‐loop plastic recycling approach.

## Results and Discussion

### Isolation of polyester‐hydrolyzing enzymes from compost metagenomes

We expected plant composts which are habitats for thermophilic microorganisms involved in the degradation of plant polymers such as cutin to be valuable sources of polyester‐hydrolyzing enzymes.[^24^, ^25^, ^26^, ^27^, ^28^] By using metagenomic methods we could also access relevant enzymes from those microorganisms which cannot be cultivated under laboratory conditions.[^26^, ^29^, ^30^] Metagenomes were collected from different compost sites located in Leipzig, Germany (Table S1). A pair of degenerate primers was designed to amplify DNA coding for polyester‐hydrolyzing enzymes. A consensus sequence derived from 54 confirmed or putative hydrolase genes was used to design primers allowing the amplification of mature protein coding sequences (Table S2). Previous studies have isolated similar enzymes from metagenomes using degenerate primers for the conserved serine hydrolase motif GXSXG, coupled with an inverse PCR step,^[26]^ screening of fosmid libraries,[^27^, ^29^] or metagenomic mining.[^28^, ^31^] In our approach, amplicons were directly cloned into an *E. coli* expression system and screened on agar plates containing tributyrin as substrate to detect ester hydrolase activity. Positive clones were further probed with polycaprolactone and PET nanoparticles as enzyme substrates (Table S3). Seven enzymes with polyester‐hydrolyzing activity (PHL, Polyester Hydrolases Leipzig) were isolated. PHL1 could be assigned to *Actinomadura hallensis* (GenBank: TQM71194.1). The other PHL showed 65.7 % to 91.5 % sequence identity with known hydrolases (Table S4). All of them contained the GXSXG box, a conserved Ser‐Asp‐His triad and an alpha/beta‐hydrolase fold. In comparison with previously characterized polyester‐hydrolyzing enzymes, several amino acids of the putative substrate binding site were exclusively found in the PHL enzymes. PHL7 has a leucine at position 210, whereas other known type I polyester hydrolases harbor a phenylalanine. PHL4 contained the sequence GWSWG instead of GHSMG flanking the catalytic serine and an unusual oxyanion hole composition (W132 instead of M132; see the Supporting Information, Figure S1).

**Figure 1:**
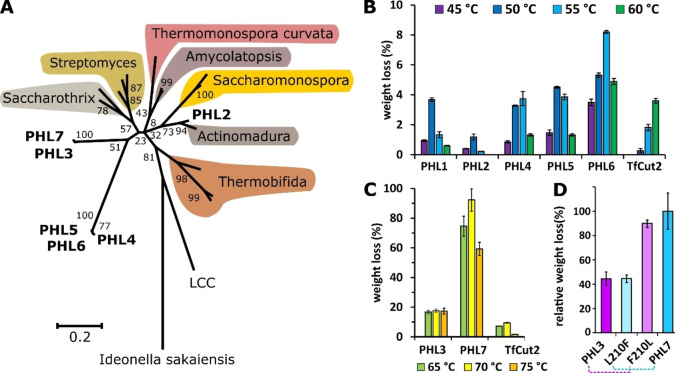
Characterization of PHL enzymes: (A) Phylogenetic tree of PHL and other confirmed or putative polyester hydrolases from Actinobacteria with reference to the metagenomic LCC and *Ideonella sakaiensis*. (B) Weight loss of amorphous PET film determined after a reaction time of 24 h at different temperatures with PHL1, PHL2, PHL4, PHL5, and PHL6 compared to TfCut2. (C) Weight loss of amorphous PET films determined after a reaction time of 24 h at different temperatures with PHL3 and PHL7 compared to TfCut2. (D) Relative weight loss of amorphous PET films determined after a reaction time of 24 h at 70 °C with PHL3, PHL7 (100 %), and the corresponding variants F210L and L210F.

A phylogenetic tree was constructed to illustrate the sequence similarities (Figure 1A). Whereas PHL2 clustered in the genus *Actinomadura*, the other PHL formed separate clusters and showed highest sequence similarities with dienelactone hydrolases and cutinases from different Actinomycetales. The DNA fragments had high GC contents between 69 % and 71 %. In comparison, LCC clustered between the phylum of actinobacteria and proteobacteria. The *Ideonella sakaiensis* PETase as a representative of the phylum Proteobacteria showed the largest distance to the PHL enzymes.

### PHL3 and PHL7 show high PET‐hydrolyzing activity

In a first screening, the isolated enzymes were expressed in *E. coli* and partially purified by IMAC chromatography. PHL3 and PHL7 exhibited the highest initial hydrolysis rates of amorphous PET films at reaction temperatures up to 80 °C (Figure S2A). Whereas PHL3 and PHL7 also caused a high weight loss of amorphous PET films, the other PHL were much less active in a similar range as the previously reported polyester hydrolase TfCut2^[32]^ (Figure 1B,C).

### Leucine at position 210 is responsible for the high activity of PHL7 compared to PHL3

A comparison of the amino acid sequences of PHL3 and PHL7 indicated differences in only four positions (A2, L210, D233, S255 in PHL7). Leucine 210 has been previously suggested to contribute to the binding of the polymer substrate to the enzyme.[^16^, ^20^, ^22^] A substitution of phenylalanine at this position with alanine, tryptophane or isoleucine has resulted in variants with higher PET‐hydrolyzing activity.[^16^, ^22^] To explore the reason for the considerably higher activity of PHL7, we exchanged leucine 210 in PHL7 with phenylalanine. As a result, the activity of the variant dropped to the level of PHL3 (Figure 1D). Likewise, when we replaced phenylalanine 210 in PHL3 with leucine, the activity of the corresponding variant increased to the level of PHL7 confirming that the identity of the amino acid residue 210 is responsible for the detected higher activity of PHL7.

### Structural features of PHL7

**Figure 2:**
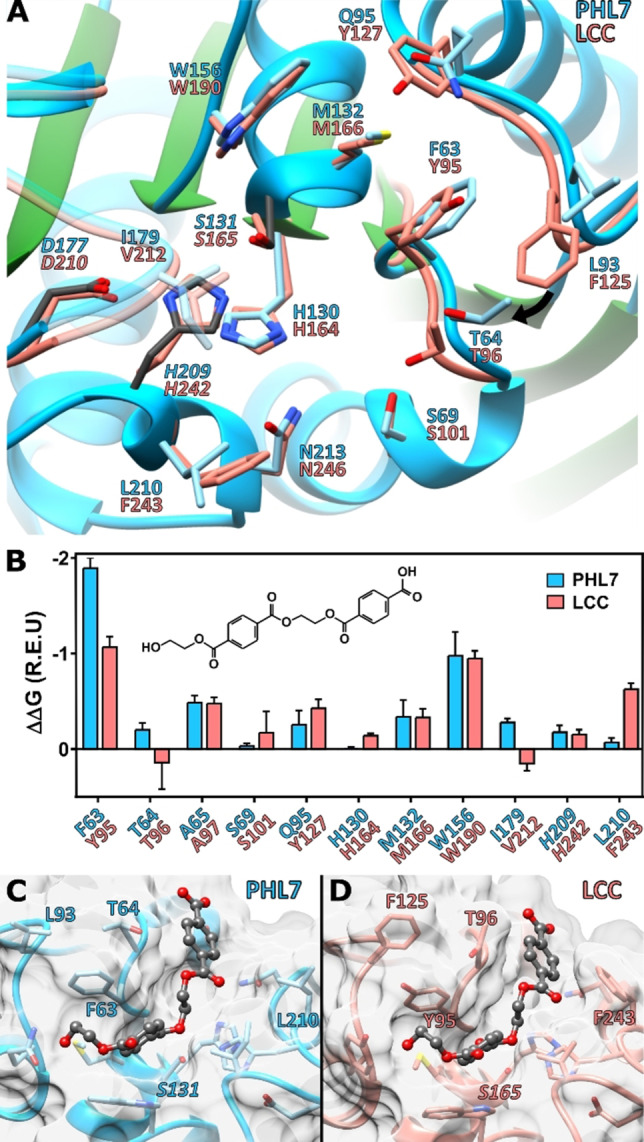
Comparison of the crystal structures of PHL7 and LCC and docking experiments: (A) Active site structures of PHL7 (chain A) and LCC. S131 adopts two different conformations, of which the most occupied is displayed. (B) Predicted per‐residue binding energy contribution based on a docking of 1,2‐ethylene monoterephthalate mono(2‐hydroxyethyl terephthalate) (EMT) in PHL7 and LCC. The best five out of 40000 complexes with the lowest interface binding energy and RMSD lower than 1.5 Å in relation to the *p*‐nitrophenol and HEMT cocrystal structure of *Is*PETase are shown.^[36]^ (C,D): Lowest RMSD pose of the 0.25 % best interface binding energy complexes of EMT with PHL7 (C) and LCC (D).

The high‐resolution crystal structure of PHL7 (1.3 Å, PDB: 7NEI; Table S5), shows the typical features of the alpha/beta‐hydrolase fold superfamily with a canonical Ser‐Asp‐His catalytic triad. The overall fold of PHL7 is almost identical to the previously reported polyester hydrolase LCC (PDB : 4EB0)^[33]^ indicated by a root‐mean‐square deviation (RMSD) value of 1.08 Å for the two chains in the asymmetric unit (alignment of 253 from a total of 259 residues). The amino acids in the active site forming the substrate‐binding cavity also exhibited similarities as well as several important differences. Residues G62, T64, S69, H130, *S131*, M132, W156, *D177*, A180, *H209*, N213 (catalytic triad in italic) are conserved between PHL7 and LCC, whereas F63 (LCC: Y95), L93 (F125), Q95 (Y127), I179 (V212) differed (Figure 2A). Figure 2A also shows that the active site pocket of PHL7 is more open than that of LCC. This affects in particular T64, L93, L210 and neighboring residues. This difference is more pronounced in chain A of the two molecules in the asymmetric unit of the PHL7 crystals and it likely results from crystal packing interactions (Figure S3). However, the plasticity of these predominantly hydrophobic regions near the active site may contribute to binding of larger PET substrates and be a key for a more tolerant recognition and effective processing of PET at elevated temperatures concomitant with increased polymer chain mobility. Indeed, the importance of L210 for catalysis has already been demonstrated (see chapter above). Interestingly, narrowing the binding site in *Is*PETase by a double mutation to introduce more bulky side chains increased its activity against PET.^[34]^

### Molecular docking demonstrates differences in ligand binding between PHL7 and LCC

To determine the amino acid residues that contribute to the PET binding affinity and degradation efficiency of PHL7, we performed molecular docking experiments with MHET, TPA and 1,2‐ethylene monoterephthalate mono(2‐hydroxyethyl terephthalate) (EMT) in comparison with LCC. Previous results obtained with the polyester hydrolase *Is*PETase have indicated that the per‐residue energetic contributions to binding affinity are in good agreement with the catalytic contributions of active site residues towards PET hydrolysis.^[35]^ Ligand RMSD‐based clustering for PHL7 showed that 29 out of 100 of the lowest binding energy EMT docking poses positioned a terephthalic ring in the same groove as seen for *p*‐nitrophenol and 4‐methyl terephthalate (HEMT) in solved structures of *Is*PETase (Figures S4 and S5).^[36]^ A similar EMT binding conformation was found in 19 complexes with LCC (Table S6). Further analysis of the five best EMT‐bound complexes of LCC and PHL7 (Figure 2C,D) demonstrated that their binding energies were similar (−11.9±1.0 and −12.0±0.6 Rosetta Energy Units (REU) for PHL7 and LCC, respectively), but the specific per‐residue contributions to ligand binding varied between these enzymes (Figure 2B). Energetic analysis of the five best docking poses for MHET (Figures S6 and S7) showed that its binding was more favored in PHL7 (−10.7±0.4 REU) than in LCC (−9.2±0.2 REU). The same phenomenon was observed for TPA (Figures S6 and S7; −10.0±0.4 and −9.0±0.3 REU for PHL7 and LCC, respectively). Interestingly, the per‐residue contribution of F63 in subsite I towards MHET and TPA binding was lower than for EMT, and similar to the energetic contribution of Y95 in LCC.

Residue F63 in PHL7, which conforms with the aromatic clamp of subsite I of polyester hydrolases,^[37]^ contributed almost twice the binding energy than the equivalent residue Y95 in LCC, whereas no differences were observed for the aromatic clamp residue W156 (W190 in LCC). Other subsite I residues, such as the contiguous T64 and also I179 gave a small contribution to EMT binding not found in LCC. In contrast, residues near or within subsite II in LCC (S101, Y127, H242, F243) gave a higher contribution to EMT binding than the equivalent residues in PHL7 (S69, Q95, H209, L210). Among them, residue F243 (LCC), which is characteristically replaced by serine in *Is*PETase^[35]^ and other Type IIb polyester hydrolases^[37]^ and is also replaced by L210 in PHL7, is one of the three residues that contributes most of the binding energy in LCC but has a negligible contribution towards EMT binding in PHL7. These results suggest that the phenylalanine/leucine replacement could be partially responsible for the changes in per‐residue binding energy contributions in PHL7, similarly to what was observed for *Is*PETase by the substitution of the highly conserved phenylalanine in thermophilic enzymes by serine.^[35]^

### Thermostability of PHL7

Analysis of the thermostability of PHL7 by nano differential scanning fluorimetry showed a temperature melting point (*T*_m_) of 79.1 °C, which is 5.2 °C lower than that of LCC (Figure S8). Since the substitution F243I in LCC reduced its melting point by 3 °C, this could explain the lower thermostability of PHL7 which has a leucine in the corresponding position 210.^[16]^ PHL7 was stabilized by increasing concentrations of Ca^2+^ and Mg^2+^ ions up to a *T*_m_ of 86.1 °C and 83.7 °C at a concentration of 100 mm, respectively (Figure S8B). The crystal structure of PHL7 shows a metal ion coordinated by the side chains of Glu148 and Asp233, the carbonyl group of Phe230 and three water molecules in a distorted octahedral geometry (Figure S9). We modeled this ion as sodium due to the electron density at this position and the high concentration (100 mm) of Na^+^ in the crystallization buffer. However, the nature of this binding site appears also well suited to coordinate Ca^2+^ ions. The *T*_m_ of PHL7 was increased to 84 °C when the phosphate buffer concentration was raised from 50 mm to 1 M. The high buffer capacity was also needed to maintain the pH of the medium during the hydrolysis reaction.[^14^, ^38^] In addition, we observed that the *T*_m_ also increased to 86 °C in the presence of a PET nanoparticle suspension, confirming previous reports on a stabilizing effect of PET on LCC activity.^[33]^

### Fast hydrolysis of amorphous PET films by PHL7

**Figure 3:**
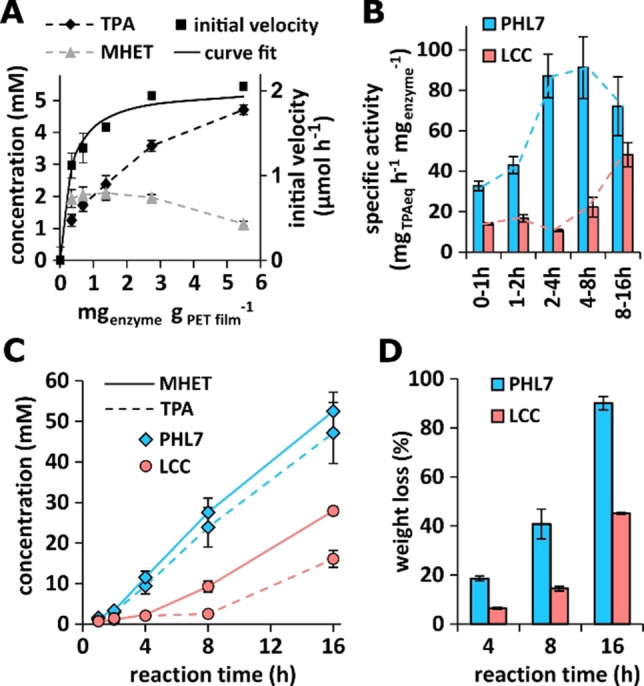
Hydrolysis of amorphous PET films by PHL7: (A) Initial reaction velocity expressed as TPA_eq_ (sum of TPA and MHET) released from G‐PET films within 1 h of reaction as a function of enzyme concentration. A curve fit of the initial velocity was performed with a Langmuir type heterogeneous kinetic model.[^14^, ^39^] (B) Specific G‐PET film hydrolysis activity of PHL7 and LCC at different reaction times with 0.6 mg_enzyme_ g_PET_^−1^. (C) TPA and MHET released from G‐PET films by PHL7 and LCC within 16 h of reaction time with 0.6 mg_enzyme_ g_PET_^−1^. (D) Comparison of the weight loss of G‐PET films after reaction times of 4, 8, and 16 h with 0.6 mg_enzyme_ g_PET_^−1^ of PHL7 and LCC.

When amorphous G‐PET films with 5–7 % crystallinity were hydrolyzed with PHL7 in concentrations ranging from 0.34 to 5.5 mg_enzyme_ g_PET_^−1^, the molar ratio of TPA : MHET detected after a reaction time of 1 h increased from 0.65 to 4.12 (Figure 3A).

This indicates that MHET initially released during the reaction was further hydrolyzed to TPA and EG in the presence of higher enzyme concentrations. From the determination of the initial velocity of the formation of TPA and MHET (TPAeq) at different enzyme concentrations, we selected 0.6 mg_enzyme_ g_PET_^−1^ as sufficient to obtain a weight loss of the PET films of >90 % within a reaction time of 16 h.

For the following PET degradation experiments, 0.6 mg_enzyme_ g_PET_^−1^ of homogeneous enzyme fractions were used and the performances of homogenous PHL7 and LCC preparations were compared (Figure S10). PHL7 released higher amounts of MHET and TPA from amorphous G‐PET films within a reaction time of 16 h compared to LCC (Figure 3B,C). A maximum specific activity of PHL7 with 91 mg TPA_eq._ h^−1^ mg_enzyme_^−1^ was determined between 4 and 8 h of reaction, compared to 48 mg TPA_eq._ h^−1^ mg_enzyme_^−1^ with LCC between 8 and 16 h. Using pulverized amorphous PET with a higher surface area, a specific activity of 82 mg TPA_eq._ h^−1^ mg_enzyme_^−1^ has previously been reported for LCC.^[16]^ In addition to TPA and MHET, small amounts of BHET, EMT and EBT (1,2‐ethylene‐bis‐terephthalate) were also detected by mass spectrometry as PET hydrolysis products of PHL7. Two further products containing one and two TPA moieties, respectively, could not be identified (Figures S11 and S12, Tables S7 and S8).

PHL7 also caused a higher weight loss from the G‐PET films (90 %±2.8 %) than LCC (45 %±0.3 %) within 16 h of reaction (Figure 3D). The films were completely degraded by PHL7 within 18 h, whereas only 73 % weight loss was observed with LCC within 24 h of reaction. PHL7 thereby outperformed other previously reported polyester hydrolases in the hydrolysis of amorphous PET films.[^14^, ^19^, ^24^, ^40^] Whereas Shirke et al. described a weight loss of G‐PET films by LCC of 25 % and of 95 % with a glycosylated LCC expressed in *Pichia pastoris* within 48 h of reaction,^[41]^ a weight loss of 42 % was reported within 24 h of reaction with an enzyme concentration of 1.1 mg_enzyme_ g_PET_^−1^.^[15]^ Sulaiman et al. reported a weight loss of an amorphous PET packaging container material using LCC of about 24 % within a reaction time of 24 h.^[33]^ Tournier et al. utilized an engineered LCC variant for the degradation of amorphous PET powder, achieving 90 % depolymerization within 10 h of reaction with 3 mg_enzyme_ g_PET_^−1^.^[16]^ The choice of enzyme production hosts and purification schemes, as well as different PET substrates and reaction conditions used in these studies are likely to account for the reported differences in LCC performance.

Biaxially oriented PET of higher crystallinity, which is for example used in PET bottles, could not be degraded by PHL7 or LCC. In agreement with this result, a thermo‐mechanical pretreatment to reduce the crystallinity of post‐consumer PET waste was required prior to the hydrolysis by LCC variants.^[16]^

### Degradation process of amorphous PET film surfaces exposed to PHL7 and LCC

**Figure 4:**
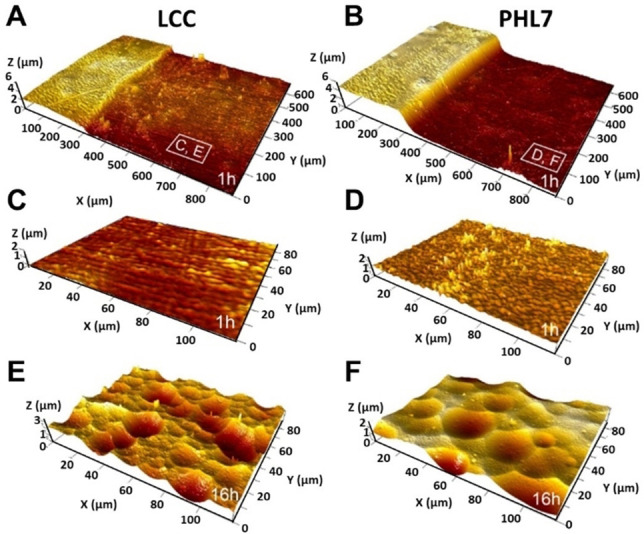
Surface topography of G‐PET films exposed to PHL7 and LCC. (A,B) Surface retreat after an exposure time of 1 h compared to a masked surface area (left part) with LCC (A) and PHL7 (B). (C–F) Topographic details of the surface sections after an exposure time of 1 h (C,D) and 16 h (E,F).

Analysis of G‐PET films by vertical scanning interferometry (VSI) after different times of exposure to PHL7 and LCC showed an irregular degradation of the film surface with the formation of pits which increased in diameter during exposure (Figure 4).

**Figure 5:**
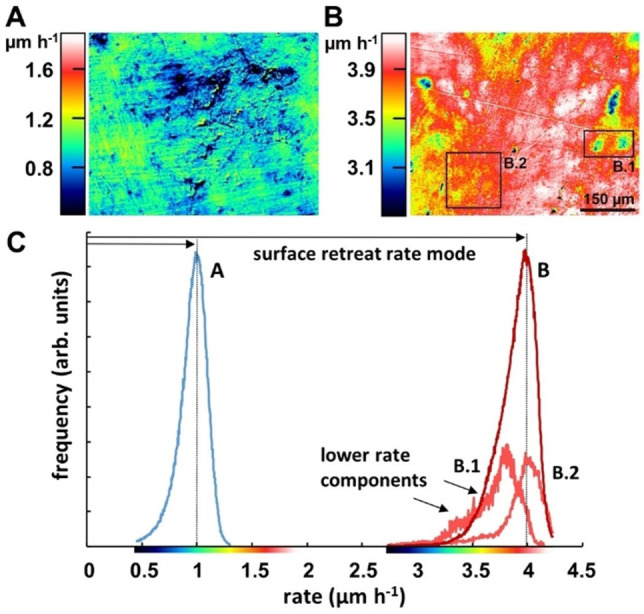
(A,B) Degradation rate maps and histograms of G‐PET films after exposure for 1 h with LCC (A) and PHL7 (B). (C) Rate distribution curves of (A) and (B) indicating different retreat rate modes of 1.0 μm h^−1^ for LCC compared to 4.0 μm h^−1^ for PHL7. Although both rate histograms are moderately skewed left, the degradation rate distribution observed with PHL7 (B) shows a larger variability of the lower rate portions, exemplified by the map and rate histograms of sections B.1 and B.2. For better visibility, the graphs of B.1 and B.2 are vertically magnified.

The corresponding degradation rate maps and histograms revealed a heterogeneous distribution of the degradation rates with a larger distribution range for PHL7 (1.5 μm h^−1^) compared to LCC (0.85 μm h^−1^). PHL7 showed a 4‐fold higher degradation rate compared to LCC, resulting in a substantial surface degradation already after an exposure time of 1 h (Figure 5).

The underlying surface images (Figure S14) and the recorded degradation rates in the exposure periods 0 h to 1 h and 1 h to 16 h (Figure S15) indicated a progressive acceleration of the degradation process by both enzymes. PHL7 showed a degradation rate of 6.7 μm h^−1^ after 16 h of exposure compared to 3 μm h^−1^ in the case of LCC.

Scanning electron microscopic analysis of the eroded PET films exposed to the two polyester hydrolases confirmed the formation of pits on the eroded surfaces (Figure S16). On the films exposed to LCC, particles and needle‐shaped structures were detected on the surface.

### Recycling of post‐consumer PET thermoform packaging by PHL7

PET thermoform packaging made of amorphous or recycled PET is increasingly used to produce clamshells, trays, boxes and tubs. With their recyclability and high versatility in the processing, PET thermoforms represent a compelling sustainable packaging solution for food products with an estimated global market size of more than 34 billion EUR.^[42]^ Post‐consumer thermoform PET packaging could be readily hydrolyzed by PHL7 without any pretreatments of the material. Four types of clamshell food containers (Table S9) were completely degraded within 24 h using 0.6 mg_enzyme_ g_PET_^−1^ at 70 °C. Similar thermoform packaging could only be partially degraded by the polyester hydrolase TfCut2 even though this enzyme completely dissolved amorphous G‐PET films of similar crystallinity within 120 h of reaction.^[24]^ The efficient degradation of amorphous PET converted from post‐consumer PET bottles by a LCC variant has also been demonstrated recently.^[16]^

As a proof of concept of a closed‐loop biocatalytic recycling, we enzymatically degraded a whole clamshell container (17.43 g) cut into flakes of approximately 3×3 cm^2^ without any further pretreatment within 48 h of reaction at 60 °C employing 0.63 mg_enzyme_ g_PET_^−1^ PHL7 at the start of the reaction and a second addition of 0.52 mg_enzyme_ g_PET_^−1^ after 24 h. An almost complete dissolution corresponding to a weight loss of 98.6 % was achieved with only a few fragments of cutting edges remaining (Figures S13 and S17). The released TPA was separated from the hydrolysate by precipitation with concentrated HCl. The purity of the TPA preparation was confirmed by HPLC and ^1^H NMR analysis (Figures S18 and S19). TPA with a purity of 94 %±1.3 % was obtained corresponding to 84.7 % of the theoretical yield. The enzymatically produced TPA was used to synthesize virgin PET with properties comparable to a commercial amorphous PET sample. Analysis by ^1^H NMR spectroscopy indicated an average degree of polymerization of 151, corresponding to an average molecular weight of 29835 g mol^−1^ (Figure S20). Analysis of the synthesized PET by Fourier‐transform infrared spectroscopy indicated the integrity of the synthesized PET (Figure S21).

Confirming these results, it has been recently demonstrated that PET synthesized with TPA obtained by enzymatic hydrolysis of post‐consumer PET could be used to make PET bottles with properties comparable to those made from fossil‐based feedstocks.^[16]^ Although petrochemical EG was used in the PET polycondensation reaction, the process can be further developed to also employ the EG produced by the enzymatic hydrolysis which could be recovered by distillation. As an alternative, EG derived from biomass could also be envisaged for producing recycled PET without the use of any petrochemicals.^[43]^

Thus, by employing powerful enzymes such as PHL7 it is possible to directly recycle post‐consumer thermoform PET packaging in a closed‐loop process with a low carbon footprint and without the use of petrochemicals, realizing a sustainable recycling process of an important PET plastic waste stream.

## Conclusion

A degenerate primer method was found to be useful for the isolation of novel metagenomic polyester hydrolases from plant composts. One of the enzymes, PHL7, hydrolyzed complete amorphous PET films with high efficiency, outperforming previously reported polyester hydrolases. The gradual surface degradation process could be monitored in detail by vertical scanning interferometry. Structural analysis of the enzyme indicated that leucine at position 210 contributed to the high PET hydrolytic activity compared to other polyester hydrolases. PHL7 could be utilized for the rapid and complete degradation of post‐consumer thermoform PET packaging at low temperatures in an aqueous reaction system. PET packaging flakes were directly degraded without the need of energy‐consuming grinding, melting, and extrusion steps thus contributing to a low carbon footprint of the process. Virgin PET with properties comparable to commercial samples made from petrochemicals could be synthesized using TPA recovered from the enzymatic PET hydrolysis process.

## Experimental Section

### Enzymes, polyester substrates and chemicals

Primers were synthesized by Metabion International AG (Planegg, Germany). Tributyrin was purchased from Carl Roth (Karlsruhe, Germany) and polycaprolactone flakes were obtained from Fluka (Sigma‐Aldrich, St. Louis, USA). Amorphous polyethylene terephthalate (PET) films (G‐PET, ES301445, thickness 250 μm) and biaxially oriented PET films (ES301450, thickness 250 μm) were from Goodfellow GmbH (Bad Nauheim, Germany). All other chemicals were obtained from Carl Roth GmbH+Co. KG (Karlsruhe, Germany) and Gruessing GmbH Analytica (Filsum, Germany) at the highest purity available.

### Primer design

The gene sequences of 54 putative or confirmed hydrolases were obtained from the gene database of the National Center for Biotechnology Information (NCBI, Bethesda, USA; Table S2). The consensus sequence was obtained with GeneFisher^[44]^ and was used to derive a degenerate primer pair with the sequence: Fw 5′‐ATG GMS AAC CCS TAC GAG CGC GG‐3′ and Rev 5′‐GWR SGG GCA GKT GSM SCG GTA CT‐3′.

### Collection and storage of the compost samples

Three different plant waste compost sites located in Leipzig, Germany, were selected to collect samples. The collection sites included a compost (about 2 years old) located at the Botanical Garden, Leipzig University, as well as two further composts (about 2 years and 6 months old) located at the South Cemetery, Leipzig. Samples were collected at six different positions in a depth of 50 to 70 cm beneath the surface of each compost pile. The six samples from each compost site were mixed in equal proportions and stored in sterile tubes at −20 °C. The core temperature, moisture content and pH of each compost pile was recorded (Figure S1).

### Isolation of metagenomic DNA, construction of the clone library and identification of polyester hydrolases

The mixed samples of the three compost sites were used to isolate metagenomic DNA as described elsewhere^[45]^ as well as by using the PowerMax Soil DNA Isolation Kit (Mo Bio Laboratories, Inc., Carlsbad, CA, USA). If residual humic acids were present in the isolated DNA samples, a second purification step using Chroma Spin+TE‐1000 columns (Clontech Laboratories Inc., Mountain View, CA, USA) was performed.

The isolated metagenomic DNA was used as a template for PCR reactions applying the degenerate primer pair. Dimethyl sulfoxide (5 %) was added to the PCR reaction mixture to facilitate the amplification of sequences with a high GC content. The following PCR program was used: 10 min at 95 °C, 34 cycles of 30 s at 95 °C, 30 s at 57.5 °C and 2 min at 72 °C, followed by a single step at 72 °C for 10 min. The resulting PCR products with a size of approximately 800 bp were extracted from an agarose gel and cloned into One Shot TOP10 *Escherichia coli* using the pBAD TOPO TA Expression Kit (Thermo Fischer Scientific, Waltham, MA, USA) according to the manufacturer's instructions.

For each compost site, 768 single colonies were screened on turbid lysogeny broth (LB) agar plates containing 100 μg mL^−1^ ampicillin, 0.2 % L‐arabinose and 1 % tributyrin. Clones forming clearing zones were further screened on turbid LB agar plates containing 100 μg mL^−1^ ampicillin, 0.2 % L‐arabinose, 0.5 mg mL^−1^ polycaprolactone dissolved in acetone or 0.4 mg mL^−1^ PET nanoparticles. The PET nanoparticle suspension was prepared as described previously.^[46]^

Recombinant genes coding for polyester‐hydrolyzing enzymes were sequenced by Seqlab Sequence Laboratories (Microsynth AG, Balgach, Switzerland) and identified using the NCBI protein database.

### Phylogenetic analysis of Polyester Hydrolases Leipzig (PHL)

The amino acid sequences of the isolated PHL were aligned with 16 trimmed (starting at residue 3 of PHL7) confirmed or putative polyester hydrolases (accession numbers: A0A0K8P6T7, ADV92527, AFA45122, BAO42836, CBY05530, CDN67545, G9BY57, TQM71194, WP 005465756, WP 024756907, WP 030875850, WP 033432831, WP 106615941, WP 123685830, WP 126889148, WP 191248857) using the ClustalW algorithm implemented in MEGA X.^[47]^ The trimmed alignment was used to create a phylogenetic tree with MEGA X. The tree was generated by the maximum likelihood method with 1500 bootstrap replicates and a LG+G substitution model.

### Expression and purification of PHL

In a first screening, the isolated PHL were expressed and partially purified by Ni‐NTA chromatography as described previously.^[48]^ As a reference, the gene encoding the polyester hydrolase TfCut2 from *Thermobifida fusca* KW3 (ENA: FR727681.1) was inserted into the pBAD vector for recombinant expression in *E. coli* One Shot TOP10. For the detailed characterization of PHL7 and comparison with LCC, the pET‐26b(+) vectors harboring the PHL7 and LCC coding sequence were expressed in *E. coli* BL21 (NEB, Ipswich, MA, USA) at 37 °C, 150 rpm and 30 μg ml^−1^ kanamycin in 2 L LB medium (4×0.5 L in 2 L flasks) up to an optical density at 600 nm of 0.6. Subsequently, the cultures were cooled to 18 °C and induced with 0.1 mm IPTG and further cultured at 18 °C for 16 h at 60 rpm. Cells were harvested and then disrupted by sonification. The supernatant obtained after centrifugation at 24000×g for 60 min was sterile‐filtered and used for subsequent purification on a Äkta purifier system (Cytiva, Marlborough, MA, USA). Buffer A consisted of 50 mm phosphate, 200 mm NaCl, pH 7.4 and for buffer B, 250 mm imidazole was added to buffer A. Ni‐NTA chromatography was performed with a 5 mL HisTrap FF column (Cytiva, Marlborough, MA, USA) with a flow rate of 5 mL min^−1^. The column was equilibrated with 5 column volumes (CV) 8 % B prior to sample injection (60 to 80 mL). The column was washed with 5 CV 8 % B and a step elution with 5 CV 16 % B and 7 CV 100 % B was performed. The fraction containing the recombinant protein was collected and incubated at 62 °C for 30 min. After centrifugation, the supernatant was concentrated with an Amicon 10 kDa cutoff ultrafiltration filter (Merck KGaA, Darmstadt, Germany). Size exclusion chromatography with a Hiload 26/60 Superdex 200 pg column (Cytiva, Marlborough, MA, USA) with buffer A yielded homogeneous enzyme preparations (Figure S10).

### Determination of the thermal stability of PHL

Reaction vials containing partially purified enzyme preparations (33.3 μg mL^−1^) in 1 m potassium phosphate buffer (pH 8.0) were incubated at 65, 70 and 75 °C for 24 h. A control sample was stored at 4 °C. The residual enzyme activity was determined with polycaprolactone nanoparticles as substrate at 50 °C using a PowerWave XS plate reader at 600 nm (BioTek Instruments, Inc., Winooski, VT, USA). The PCL nanoparticles were prepared as described previously.^[48]^ An enzyme concentration of 5 μg mL^−1^ in 1 m potassium phosphate buffer (pH 8.0) was used. Purified PHL7 (0.2 mg mL^−1^) was incubated in 50 mm phosphate buffer between 65 °C and 74 °C for 4 h, 16 h and 24 h in an ep gradient S thermocycler (Eppendorf, Hamburg, Germany). The residual activity was determined with *p*‐nitrophenol butyrate (*p*NPB) as substrate at 25 °C in 100 mm phosphate buffer, pH 7.5 containing 0.5 mm *p*NPB and 10 % ethanol. The absorbance of *p*‐nitrophenol released was monitored at 405 nm. Measurements were performed with a Synergy Mx microplate reader (BioTek Instruments Inc., Winooski, VT, USA). Mean values ±S.D. for *n*=3 are shown.

### Hydrolysis of amorphous PET films by PHL

For the determination of the initial hydrolysis rates of PHL candidates in the first screening process, amorphous G‐PET films (9 cm^2^, about 150 mg) were added to reaction vials containing 0.11 mg_enzyme_ g_PET_^−1^ of the partially purified enzyme preparation and 1 m potassium phosphate buffer (pH 8.0) in a total volume of 1.8 mL. The vials were incubated at 40 °C to 85 °C on a thermo shaker (1000 rpm) for 1 h. Released hydrolysis products were quantified by HPLC.^[49]^ The sum (TPA_eq_) of the released soluble products terephthalic acid (TPA), mono(2‐hydroxyethyl) terephthalate (MHET) and bis(2‐hydroxyethyl) terephthalate (BHET) was used to determine the initial hydrolysis rates. For analyzing reactions with different concentrations of enzyme, a reaction volume of 0.35 mL and G‐PET films with 6 mm diameter (0.62 cm^2^, about 7.1 mg) were used as substrate. The hydrolysis products TPA and MHET were determined after 1 h and 2 h of reaction with enzyme concentrations of 0.34, 0.69, 1.37, 2.75 and 5.49 mg_enzyme_ g_PET_^−1^ by HPLC (see section 1.13). A curve fit was performed as previously described.[^14^, ^39^] For the determination of the weight loss of G‐PET films following enzymatic degradation, the films (3×0.5 cm, about 45 mg) were added to reaction vials containing 1.18 mg_enzyme_ g_PET_^−1^ of partially purified enzyme preparations or 0.55 mg_enzyme_ g_PET_^−1^ of a homogeneous enzyme preparations. The reactions were performed in 1 m potassium phosphate buffer (pH 8.0) in a total volume of 1.8 mL. For the initial screening of the PHL, the vials were incubated at 45 °C to 75 °C on a thermo shaker (1000 rpm) for 24 h. Further studies with homogeneous preparations of PHL7 and LCC (0.55 mg_enzyme_ g_PET_^−1^) were performed at 70 °C at 700 rpm. Weight loss of the G‐PET films was determined gravimetrically as described previously.^[50]^ Mean values ±S.D. for *n*=3 are shown.

### Vertical Scanning Interferometry

Surface topographies of amorphous PET films exposed to PHL7 and LCC were measured using an S neox 3D optical profiler (Sensofar Metrology, Barcelona, Spain), both in the vertical scanning interferometry (VSI) mode and in the confocal profiling mode, the latter based on spatially resolved focus detection. VSI was performed with Mirau objectives (Table S10) using white‐light illumination.^[51]^ For the construction of the rate maps and rate spectra calculations, sample surface topographies were analyzed before and after each exposure period of the films with the enzymes. G‐PET films were fixed on glass plates with epoxy resin and a part of the film surface was masked with Teflon tape as control. The obtained topography datasets were processed with the software SPIP (version: 6.7.3; Image Metrology A/S, Hørsholm, Denmark). Using the inert mask as a height reference, the individual datasets were correlated and subtracted from each other. The height change (d*z*) at a given surface point (*x*,*y*) over the exposure time (d*t*) was determined. The velocity of height changes (d*z*/d*t*) indicating the material flux at each point of the surface and the overall dataset including all (*x*,*y*) points were used to construct material flux maps. A histogram analysis of the rate maps resulted in a material flux or reaction rate spectrum that provided information about the frequency of rate contributors to the overall rate.^[52]^

### Molecular Docking experiments with PHL7 and LCC

To further explore the binding poses of PET onto the active site of PHL7 and LCC, the soluble substrate analog EMT was used for molecular docking with Rosetta3.[^35^, ^53^] To further analyze the binding mode of the terephthalic ring in the active site of the enzymes, molecular docking of MHET and TPA was also performed. The conformational diversity of the ligands was represented by creating 277, 52 and 3 conformers for EMT, MHET and TPA, respectively, using the confab package of Open Babel.^[54]^ The conformers were generated based on an energy cutoff of 50 kcal mol^−1^ and an RMSD cutoff of 1.3 Å, 0.4 Å and 0.4 Å for EMT, MHET and TPA, respectively. For the docking procedure, 40000 enzyme‐substrate and enzyme‐product complex structures were generated with a custom Rosetta3 XML script enabling backbone and side‐chain flexibility as previously described.^[35]^ The top 100 docking poses for all enzyme‐ligand pairs were subjected to pairwise ligand Root Mean Square Deviation (RMSD) calculation using DockRMSD,^[55]^ to generate a distance matrix for unsupervised exploratory hierarchical clustering analysis. The ligand RMSD matrix was employed to build an euclidean distance matrix using the dist() function built in R stats^[56]^ package and the Ward.D2 method was used to cluster the distance matrix. For visualization of the clustering results, we employed fviz_cluster function included in factoextra^[57]^ and ggplot2.^[58]^ The cluster that best represented the optimal binding poses of EMT, MHET and TPA into the active sites of PHL7 and LCC was determined by visually assessing that the terephthalic ring of the molecules occupied the same site as the esterase product *p*‐nitrophenol (PDB 5XH2) and the MHET analog 1‐(2‐hydroxyethyl) 4‐methyl terephthalate (HEMT, PDB 5XH3) in available crystal structures of a double mutant of the polyester hydrolase *Is*PETase.^[36]^ The lowest binding energy complex for each enzyme‐ligand pair was selected along with four additional complexes with lowest ligand RMSD for decomposition of the per‐residue energetic contributions to ligand binding using Rosetta3 DDG mover.^[59]^

### Degradation of post‐consumer PET packaging by PHL7 and recovery of TPA

A homogeneous PHL7 preparation (0.6 mg_enzyme_ g_PET_^−1^) in 1 m phosphate buffer was used to degrade flakes (3×0.5 cm^2^, ∼40 to 50 mg) of different thermoform PET clamshell containers at 70 °C for 24 h (Table S9). The crystallinity of the PET samples was determined by DSC as described previously.^[24]^ The weight loss of the films was determined by gravimetric analysis. For the recovery of TPA from enzymatic PET hydrolysate, the labels were removed from a whole PET clamshell container (17.43 g) from Guillin (Groupe Guillin, Ornans, France). The container was washed with 0.5 % aqueous SDS, deionized water and 70 % ethanol, dried over night at 50 °C and cut into 3×3 cm flakes. The flakes were added to 1 m potassium phosphate buffer (pH 8) in a total volume of 784 mL containing 0.63 mg_enzyme_ g_PET_^−1^ of a homogeneous PHL7 preparation for 48 h with shaking at 60 °C. An additional 0.52 mg_enzyme_ g_PET_^−1^ was added after 24 h of the reaction. The remaining solids were washed with 0.5 % SDS solution followed by 70 % ethanol solution prior to determination of the residual weight. The hydrolysate was filtered (0.22 μm MCE membrane, MF‐Millipore, Merck KGaA, Darmstadt, Germany), 0.5 % aqueous SDS was added and filtered again. The pH of the filtrate was then adjusted to pH 11 with NaOH to hydrolyze remaining MHET and subsequently adjusted to pH 2 with 37 % HCl. The precipitate obtained was washed several times with deionized water and the precipitation step was repeated to obtain a purified TPA preparation.

### Synthesis of PET

The purified TPA was used for PET synthesis. A two‐step polymerization of PET with the purified TPA and EG was performed.^[60]^ The first step was an esterification reaction with TPA and EG to obtain BHET followed by a polycondensation reaction of BHET. The weight ratio of EG : TPA was 1.5 Zinc acetate (150 ppm), sodium acetate (150 ppm) and antimony oxide (500 ppm) were added as catalysts in the polycondensation reaction. TPA, EG and catalysts were added to a reactor and mixed overnight. The esterification was performed at 190 °C for 8 h under stirring. The reactor was then heated up to 220 °C for 2 h and the reaction mixture became transparent. The temperature was increased to 270–280 °C and a 3 mbar vacuum was applied for 3 h to remove the EG. The synthesized PET was of white and light‐yellow color and was further characterized by FT IR and ^1^H NMR.

## Conflict of interest

The authors declare no conflict of interest.

## Supporting information

As a service to our authors and readers, this journal provides supporting information supplied by the authors. Such materials are peer reviewed and may be re‐organized for online delivery, but are not copy‐edited or typeset. Technical support issues arising from supporting information (other than missing files) should be addressed to the authors.

Supporting Information

## References

[1] R. Geyer , J. R. Jambeck , K. L. Law , Sci. Adv. 2017, 3, e1700782. 28776036 10.1126/sciadv.1700782 PMC5517107

[2a] Y. Chae , Y.-J. An , Environ. Pollut. 2018, 240, 387–395; 29753246 10.1016/j.envpol.2018.05.008

[2b] F. M. Windsor , I. Durance , A. A. Horton , R. C. Thompson , C. R. Tyler , S. J. Ormerod , Global Change Biol. 2019, 25, 1207–1221; 10.1111/gcb.14572 PMC6850656 30663840

[2c] W. W. Y. Lau , Y. Shiran , R. M. Bailey , E. Cook , M. R. Stuchtey , J. Koskella , C. A. Velis , L. Godfrey , J. Boucher , M. B. Murphy , R. C. Thompson , E. Jankowska , A. Castillo , T. D. Pilditch , B. Dixon , L. Koerselman , E. Kosior , E. Favoino , J. Gutberlet , S. Baulch , M. E. Atreya , D. Fischer , K. K. He , M. M. Petit , U. R. Sumaila , E. Neil , M. V. Bernhofen , K. Lawrence , J. E. Palardy , Science 2020, 369, 1455–1461. 32703909 10.1126/science.aba9475

[3] J. P. Jog , J. Macromol. Sci. Polym. Rev. 1995, 35, 531–553.

[4] B. Kuczenski , R. Geyer , Resour. Conserv. Recycl. 2010, 54, 1161–1169.

[5] W. Zimmermann , Philos. Trans. R. Soc. A 2020, 378, 20190273. 10.1098/rsta.2019.0273 PMC7422893 32623985

[6] T. Tiso , T. Narancic , R. Wei , E. Pollet , N. Beagan , K. Schröder , A. Honak , M. Jiang , S. T. Kenny , N. Wierckx , R. Perrin , L. Avérous , W. Zimmermann , K. O′Connor , L. M. Blank , Metab. Eng. 2021, 66, 167–178. 33865980 10.1016/j.ymben.2021.03.011

[7a] F. Kawai , ChemSusChem 2021, 14, https://doi.org/10.1002/cssc.202100740;33053253

[7b] A. M. de Castro , A. Carniel , D. Stahelin , L. S. Chinelatto Jr. , H. d A Honorato , S. M. C. de Menezes , Process Biochem. 2019, 81, 85–91.

[8] F. Kawai , T. Kawabata , M. Oda , Appl. Microbiol. Biotechnol. 2019, 103, 4253–4268. 30957199 10.1007/s00253-019-09717-y PMC6505623

[9a] R. Francis , Recycling of Polymers: Methods, Characterization and Applications, 1 ^st^ ed. (Ed.: R. Francis ), Wiley-VCH, Weinheim 2016;

[9b] D. Paszun , T. Spychaj , Ind. Eng. Chem. Res. 1997, 36, 1373–1383;

[9c] K. Ragaert , L. Delva , K. van Geem , Waste Manage. 2017, 69, 24–58. 10.1016/j.wasman.2017.07.044 28823699

[10a] W. Zimmermann , S. Billig , Adv. Biochem. Eng./Biotechnol. 2011, 125, 97–120; 10.1007/10_2010_87 21076908

[10b] R. Wei , W. Zimmermann , Microb. Biotechnol. 2017, 10, 1302–1307; 28401691 10.1111/1751-7915.12714 PMC5658586

[10c] C. M. Carr , D. J. Clarke , A. D. W. Dobson , Front. Microbiol. 2020, 11, 571265; 33262744 10.3389/fmicb.2020.571265 PMC7686037

[10d] A. Berselli , M. J. Ramos , M. C. Menziani , ChemBioChem 2021, 22, 2032–2050. 33470503 10.1002/cbic.202000841

[11a] D. Ribitsch , A. Hromic , S. Zitzenbacher , B. Zartl , C. Gamerith , A. Pellis , A. Jungbauer , A. Łyskowski , G. Steinkellner , K. Gruber , R. Tscheliessnig , E. Herrero Acero , G. M. Guebitz , Biotechnol. Bioeng. 2017, 114, 2481–2488; 28671263 10.1002/bit.26372

[11b] U. Thumarat , T. Kawabata , M. Nakajima , H. Nakajima , A. Sugiyama , K. Yazaki , T. Tada , T. Waku , N. Tanaka , F. Kawai , J. Biosci. Bioeng. 2015, 120, 491–497; 25910960 10.1016/j.jbiosc.2015.03.006

[11c] I. Kleeberg , K. Welzel , J. Vandenheuvel , R.-J. Müller , W.-D. Deckwer , Biomacromolecules 2005, 6, 262–270; 15638529 10.1021/bm049582t

[11d] S. Chen , L. Su , S. Billig , W. Zimmermann , J. Chen , J. Wu , J. Mol. Catal. B 2010, 63, 121–127;

[11e] S. Chen , L. Su , J. Chen , J. Wu , Biotechnol. Adv. 2013, 31, 1754–1767; 24055682 10.1016/j.biotechadv.2013.09.005

[11f] E. Nikolaivits , M. Kanelli , M. Dimarogona , E. Topakas , Catalysts 2018, 8, 612.

[12a] N. M. Alves , J. F. Mano , E. Balaguer , J. M. Meseguer Dueñas , J. L. Gómez Ribelles , Polymer 2002, 43, 4111–4122;

[12b] R.-J. Mueller , Process Biochem. 2006, 41, 2124–2128;

[12c] E. Marten , R.-J. Müller , W.-D. Deckwer , Polym. Degrad. Stab. 2005, 88, 371–381.

[13] R.-J. Müller , H. Schrader , J. Profe , K. Dresler , W.-D. Deckwer , Macromol. Rapid Commun. 2005, 26, 1400–1405.

[14] Å. M. Ronkvist , W. Xie , W. Lu , R. A. Gross , Macromolecules 2009, 42, 5128–5138.

[15] R. Wei , C. Song , D. Gräsing , T. Schneider , P. Bielytskyi , D. Böttcher , J. Matysik , U. T. Bornscheuer , W. Zimmermann , Nat. Commun. 2019, 10, 5581. 31811142 10.1038/s41467-019-13492-9 PMC6897938

[16] V. Tournier , C. M. Topham , A. Gilles , B. David , C. Folgoas , E. Moya-Leclair , E. Kamionka , M.-L. Desrousseaux , H. Texier , S. Gavalda , M. Cot , E. Guémard , M. Dalibey , J. Nomme , G. Cioci , S. Barbe , M. Chateau , I. André , S. Duquesne , A. Marty , Nature 2020, 580, 216–219. 32269349 10.1038/s41586-020-2149-4

[17] S. Kato , S. Sakai , M. Hirai , E. Tasumi , M. Nishizawa , K. Suzuki , K. Takai , Microbes Environ. 2018, 33, 107–110. 29459499 10.1264/jsme2.ME17165 PMC5877337

[18] X. Xi , K. Ni , H. Hao , Y. Shang , B. Zhao , Z. Qian , Enzyme Microb. Technol. 2021, 143, 109715. 33375975 10.1016/j.enzmictec.2020.109715

[19] S. Yoshida , K. Hiraga , T. Takehana , I. Taniguchi , H. Yamaji , Y. Maeda , K. Toyohara , K. Miyamoto , Y. Kimura , K. Oda , Science 2016, 351, 1196–1199. 26965627 10.1126/science.aad6359

[20] R. Wei , T. Oeser , J. Schmidt , R. Meier , M. Barth , J. Then , W. Zimmermann , Biotechnol. Bioeng. 2016, 113, 1658–1665. 26804057 10.1002/bit.25941

[21] F. Kawai , M. Oda , T. Tamashiro , T. Waku , N. Tanaka , M. Yamamoto , H. Mizushima , T. Miyakawa , M. Tanokura , Appl. Microbiol. Biotechnol. 2014, 98, 10053–10064. 24929560 10.1007/s00253-014-5860-y

[22] M. Furukawa , N. Kawakami , A. Tomizawa , K. Miyamoto , Sci. Rep. 2019, 9, 16038. 31690819 10.1038/s41598-019-52379-z PMC6831586

[23a] M. A. M. E. Vertommen , V. A. Nierstrasz , M. van der Veer , M. M. C. G. Warmoeskerken , J. Biotechnol. 2005, 120, 376–386; 16115695 10.1016/j.jbiotec.2005.06.015

[23b] K. Herzog , R.-J. Müller , W.-D. Deckwer , Polym. Degrad. Stab. 2006, 91, 2486–2498;

[23c] F. Quartinello , S. Vajnhandl , J. Volmajer Valh , T. J. Farmer , B. Vončina , A. Lobnik , E. Herrero Acero , A. Pellis , G. M. Guebitz , Microb. Biotechnol. 2017, 10, 1376–1383. 28574165 10.1111/1751-7915.12734 PMC5658601

[24] R. Wei , D. Breite , C. Song , D. Gräsing , T. Ploss , P. Hille , R. Schwerdtfeger , J. Matysik , A. Schulze , W. Zimmermann , Adv. Sci. 2019, 6, 1900491. 10.1002/advs.201900491 PMC6662049 31380212

[25a] J. Ryckeboer , J. Mergaert , K. Vaes , S. Klammer , D. D. Clercq , J. Coosemans , D. D. Clercq , J. Cossemans , H. Insam , J. Swings , Ann. Microbiol. 2003, 53, 349–410;

[25b] R. Wei , T. Oeser , W. Zimmermann , Adv. Appl. Microbiol. 2014, 89, 267–305. 25131405 10.1016/B978-0-12-800259-9.00007-X

[26] X. Hu , U. Thumarat , X. Zhang , M. Tang , F. Kawai , Appl. Microbiol. Biotechnol. 2010, 87, 771–779. 20393707 10.1007/s00253-010-2555-x

[27] S. Sulaiman , S. Yamato , E. Kanaya , J.-J. Kim , Y. Koga , K. Takano , S. Kanaya , Appl. Environ. Microbiol. 2012, 78, 1556–1562. 22194294 10.1128/AEM.06725-11 PMC3294458

[28] D. Danso , C. Schmeisser , J. Chow , W. Zimmermann , R. Wei , C. Leggewie , X. Li , T. Hazen , W. R. Streit , Appl. Environ. Microbiol. 2018, 84, e02773–17. 29427431 10.1128/AEM.02773-17 PMC5881046

[29] A. Popovic , T. Hai , A. Tchigvintsev , M. Hajighasemi , B. Nocek , A. N. Khusnutdinova , G. Brown , J. Glinos , R. Flick , T. Skarina , T. N. Chernikova , V. Yim , T. Brüls , D. Le Paslier , M. M. Yakimov , A. Joachimiak , M. Ferrer , O. V. Golyshina , A. Savchenko , P. N. Golyshin , A. F. Yakunin , Sci. Rep. 2017, 7, 44103. 28272521 10.1038/srep44103 PMC5341072

[30] K. G. Lloyd , A. D. Steen , J. Ladau , J. Yin , L. Crosby , mSystems 2018, 3, https://doi.org/10.1128/mSystems.00055-18.

[31] M. Hajighasemi , A. Tchigvintsev , B. Nocek , R. Flick , A. Popovic , T. Hai , A. N. Khusnutdinova , G. Brown , X. Xu , H. Cui , J. Anstett , T. N. Chernikova , T. Brüls , D. Le Paslier , M. M. Yakimov , A. Joachimiak , O. V. Golyshina , A. Savchenko , P. N. Golyshin , E. A. Edwards , A. F. Yakunin , Environ. Sci. Technol. 2018, 52, 12388–12401. 30284819 10.1021/acs.est.8b04252 PMC12447631

[32] C. Roth , R. Wei , T. Oeser , J. Then , C. Föllner , W. Zimmermann , N. Sträter , Appl. Microbiol. Biotechnol. 2014, 98, 7815–7823. 24728714 10.1007/s00253-014-5672-0

[33] S. Sulaiman , D.-J. You , E. Kanaya , Y. Koga , S. Kanaya , Biochemistry 2014, 53, 1858–1869. 24593046 10.1021/bi401561p

[34] H. P. Austin , M. D. Allen , B. S. Donohoe , N. A. Rorrer , F. L. Kearns , R. L. Silveira , B. C. Pollard , G. Dominick , R. Duman , K. El Omari , V. Mykhaylyk , A. Wagner , W. E. Michener , A. Amore , M. S. Skaf , M. F. Crowley , A. W. Thorne , C. W. Johnson , H. L. Woodcock , J. E. McGeehan , G. T. Beckham , Proc. Natl. Acad. Sci. USA 2018, 115, E4350–E4357. 29666242 10.1073/pnas.1718804115 PMC5948967

[35] T. Fecker , P. Galaz-Davison , F. Engelberger , Y. Narui , M. Sotomayor , L. P. Parra , C. A. Ramírez-Sarmiento , Biophys. J. 2018, 114, 1302–1312. 29590588 10.1016/j.bpj.2018.02.005 PMC5883944

[36] X. Han , W. Liu , J.-W. Huang , J. Ma , Y. Zheng , T.-P. Ko , L. Xu , Y.-S. Cheng , C.-C. Chen , R.-T. Guo , Nat. Commun. 2017, 8, 2106. 29235460 10.1038/s41467-017-02255-z PMC5727383

[37] S. Joo , I. J. Cho , H. Seo , H. F. Son , H.-Y. Sagong , T. J. Shin , S. Y. Choi , S. Y. Lee , K.-J. Kim , Nat. Commun. 2018, 9, 382. 29374183 10.1038/s41467-018-02881-1 PMC5785972

[38a] J. Schmidt , R. Wei , T. Oeser , M. R. Belisário-Ferrari , M. Barth , J. Then , W. Zimmermann , FEBS Open Bio 2016, 6, 919–927; 10.1002/2211-5463.12097 PMC5011490 27642555

[38b] C. Gamerith , M. Vastano , S. M. Ghorbanpour , S. Zitzenbacher , D. Ribitsch , M. T. Zumstein , M. Sander , E. Herrero Acero , A. Pellis , G. M. Guebitz , Front. Microbiol. 2017, 8, 938. 28596765 10.3389/fmicb.2017.00938 PMC5443175

[39] M. Scandola , M. L. Focarete , G. Frisoni , Macromolecules 1998, 31, 3846–3851.

[40] M. Oda , Y. Yamagami , S. Inaba , T. Oida , M. Yamamoto , S. Kitajima , F. Kawai , Appl. Microbiol. Biotechnol. 2018, 102, 10067–10077. 30250976 10.1007/s00253-018-9374-x

[41] A. N. Shirke , C. White , J. A. Englaender , A. Zwarycz , G. L. Butterfoss , R. J. Linhardt , R. A. Gross , Biochemistry 2018, 57, 1190–1200. 29328676 10.1021/acs.biochem.7b01189

[42] Grand View Research, Inc.: Market Research Report, “Thermoform Packaging Market Size, Share & Trends Analysis Report”, www.grandviewresearch.com/industry-analysis/thermoform-packaging-market.

[43] J. Pang , M. Zheng , R. Sun , A. Wang , X. Wang , T. Zhang , Green Chem. 2016, 18, 342–359.

[44] R. Giegerich , F. Meyer , C. Schleiermacher , Proc. Int. Conf. Intell. Syst. Mol. Biol. 1996, 4, 68–77. 8877506

[45] J. Zhou , M. A. Bruns , J. M. Tiedje , Appl. Environ. Microbiol. 1996, 62, 316–322. 8593035 10.1128/aem.62.2.316-322.1996 PMC167800

[46] R. Wei , T. Oeser , M. Barth , N. Weigl , A. Lübs , M. Schulz-Siegmund , M. C. Hacker , W. Zimmermann , J. Mol. Catal. B 2014, 103, 72–78.

[47] S. Kumar , G. Stecher , M. Li , C. Knyaz , K. Tamura , Mol. Biol. Evol. 2018, 35, 1547–1549. 29722887 10.1093/molbev/msy096 PMC5967553

[48] R. Wei , T. Oeser , J. Then , N. Kühn , M. Barth , J. Schmidt , W. Zimmermann , AMB Express 2014, 4, 44. 25405080 10.1186/s13568-014-0044-9 PMC4231364

[49] M. Barth , T. Oeser , R. Wei , J. Then , J. Schmidt , W. Zimmermann , Biochem. Eng. J. 2015, 93, 222–228.

[50] J. Then , R. Wei , T. Oeser , M. Barth , M. R. Belisário-Ferrari , J. Schmidt , W. Zimmermann , Biotechnol. J. 2015, 10, 592–598. 25545638 10.1002/biot.201400620

[51] A. Luettge , E. Bolton , A. Lasaga , Am. J. Sci. 1999, 299, 652–678.

[52] C. Fischer , A. Luttge , Proc. Natl. Acad. Sci. USA 2018, 115, 897–902. 29339525 10.1073/pnas.1711254115 PMC5798326

[53a] A. Leaver-Fay , M. Tyka , S. M. Lewis , O. F. Lange , J. Thompson , R. Jacak , K. W. Kaufman , P. D. Renfrew , C. A. Smith , W. Sheffler , I. W. Davis , S. Cooper , A. Treuille , D. J. Mandell , F. Richter , Y. E. A. Ban , S. J. Fleishman , J. E. Corn , D. E. Kim , S. Lyskov , M. Berrondo , S. Mentzer , Z. Popović , J. J. Havranek , J. Karanicolas , R. Das , J. Meiler , T. Kortemme , J. J. Gray , B. Kuhlman , D. Baker , P. Bradley , Methods Enzymol. 2011, 487, 545–574; 21187238 10.1016/B978-0-12-381270-4.00019-6 PMC4083816

[53b] G. Lemmon , J. Meiler , Methods Mol. Biol. 2012, 819, 143–155. 22183535 10.1007/978-1-61779-465-0_10 PMC3749076

[54] N. M. O'Boyle , M. Banck , C. A. James , C. Morley , T. Vandermeersch , G. R. Hutchison , J. Cheminf. 2011, 3, 33. 10.1186/1758-2946-3-33 PMC3198950 21982300

[55] E. W. Bell , Y. Zhang , J. Cheminf. 2019, 11, 40.

[56] R Core Team, *R: A language and environment for statistical computing*. R Foundation for Statistical Computing, Vienna, Austria, R Core Team **2020**.

[57] A. Kassambara, *Factoextra*, Extract and Visualize the Results of Multivariate Data Analyses **2020**.

[58] H. Wickham , Ggplot2. Elegant Graphics for Data Analysis, Springer-Verlag New York, New York, NY 2009.

[59] N. S. Alexander , A. M. Preininger , A. I. Kaya , R. A. Stein , H. E. Hamm , J. Meiler , Nat. Struct. Mol. Biol. 2014, 21, 56–63. 24292645 10.1038/nsmb.2705 PMC3947367

[60a] V. G. Mihucz , G. Záray , Appl. Spectrosc. Rev. 2016, 51, 183–209;

[60b] A. C. Espinosa-López , C. A. Ávila-Orta , F. J. Medellín-Rodríguez , P. González-Morones , C. A. Gallardo-Vega , P. A. de León-Martínez , M. Navarro-Rosales , J. A. Valdez-Garza , Polym. Bull. 2019, 76, 2931–2944;

[60c] P. Švec , P. Hubená , Z. Růžičková , J. Holubová , M. Pouzar , J. Merna , A. Růžička , Appl. Organomet. Chem. 2016, 30, 20–25.

