# Supplementary material for: Low Carbon Footprint Recycling of Post‐Consumer PET Plastic with a Metagenomic Polyester Hydrolase
Source: ChemSusChem. 2022 Feb 10;15(9):e202101062. doi: 10.1002/cssc.202101062 (PMC9303343; doi:10.1002/cssc.202101062)
Supplement: Supplementary file 1 — Supporting Information [file CSSC-15-0-s001.pdf]

ChemSusChem

Supporting Information

Low Carbon Footprint Recycling of Post-Consumer PET Plastic with a Metagenomic Polyester Hydrolase

Christian Sonnendecker,* Juliane Oeser, P. Konstantin Richter, Patrick Hille, Ziyue Zhao, Cornelius Fischer, Holger Lippold, Paula Blázquez-Sánchez, Felipe Engelberger, César A. Ramírez-Sarmiento, Thorsten Oeser, Yuliia Lihanova, Ronny Frank, Heinz-Georg Jahnke, Susan Billig, Bernd Abel, Norbert Sträter, Jörg Matysik, and Wolfgang Zimmermann*This publication is part of a collection of invited contributions focusing on "Biocatalysis as Key to Sustainable Industrial Chemistry". Please visit to view all contributions.© 2021 The Authors. ChemSusChem published by Wiley-VCH GmbH. This is an open access article under the terms of the Creative Commons Attribution License, which permits use, distribution and reproduction in any medium, provided the original work is properly cited.

Table of Contents

Table of Contents 2
Experimental Procedures 3
Tables and Figures 4
Table S1 4
Table S2 4
Table S3 6
Table S4 7
Table S5 8
Table S6 8
Table S7 9
Table S8 9
Figure S1 11
Figure S2 12
Figure S4 14
Figure S5 15
Figure S6 16
Figure S7 16
Figure S8 17
Figure S10 17
Figure S13 19
Figure S14 20
Figure S17 22
Figure S18 23
Figure S19 24
Figure S20 25
Figure S21 26
References 27

Experimental Procedures

Site-directed mutagenesis

Site-directed mutagenesis of PHL3 and PHL7 was performed using the QuikChange Lightning Kit from Stratagene (La Jolla, CA, USA) according to the manufacturer's instructions. The following primers were used (amino acid substitutions are underlined):

Fw PHL-3 F210L 5'-GTGTTCGACACGAGGTGGCTGGCCCCG

Rev PHL-3 F210L 5'-CGGGGCCAGCCACCTCGTGTCGAACAC

Fw PHL-7 L210F 5'-GTGTTCGACACGAAGTGGCTGGCCCCG

Rev PHL-7 L210F 5'-CGGGGCCAGCCACTTCGTGTCGAACAC.

Following verification of the desired mutations by sequencing, the plasmids were transformed into chemically competent *E. coli* OneShot TOP10 cells (Thermo Fisher Scientific, Waltham, MA, USA).

Subcloning into pET-26b(+)

DNA encoding for the mature proteins PHL7 and LCC were cloned into a pET-26b(+) expression vector at the NdeI, XhoI sites utilizing an Rf-cloning strategy[1] with the following primer pairs:

Fw-pET26_PHL-7 5'-attttgtttaactttaagaaggagatatacatatgGCGAACCCGTACGAGC

Rev-pET26_PHL-7 5'-gtggtggtggtgctcgagGAACGGGCAGGTGGAG

Fw-pET26_LCC 5'-attttgtttaactttaagaaggagatatacatatgTCTAACCCGTACCAGCG

Rev-pET26_LCC 5'-gtggtggtggtgctcgagCTGGCAGTGACGGTTG

Capital letters in the primer mark the protein encoding region of the primers. The PCR was set according to the Rf-cloning prediction tool[1] and Phusion DNA-Polymerase was used according to the manufacturer's instructions (Thermo Fisher Scientific, Waltham, MA, USA). *E. coli* XL10Gold cells were transformed with the DpnI digested PCR products. The constructed plasmids were then isolated and sequenced prior to introduction into *E. coli* BL21 (DE3).

Sodium dodecyl sulfate -polyacrylamide gel electrophoresis (SDS-PAGE)

SDS-PAGE was used to monitor the purity of the enzyme samples. Protein separation was performed in a 15% polyacrylamide gel (Tris-glycine buffer). Samples (4 µg) were loaded under reducing conditions. A pre-stained molecular marker (PS10 PLUS;11-180 kDa, Gene On, Ludwigshafen, Germany) was used for size evaluation. Staining was performed with Coomassie Brilliant Blue G-250.

Determination of protein melting temperatures

Protein melting temperatures (T_m) were determined by nano differential scanning fluorimetry (nanoDSF) with a Prometheus NT.48 (Nanotemper Technologies, Munich, Germany). Measurements were performed with a heating ramp from 20°C to 95°C and a slope of 1°C min^{-1}. Mean values ± SD for n=3 are shown.

HPLC analysis of enzymatic PET hydrolysis products

HPLC analysis of hydrolysis products from experiments with homogeneous PHL7 and LCC preparations were performed with an Agilent 1100 HPLC system (Agilent Technologies Waldbronn, Germany) consisting of a G1367A well plate autosampler, G1312A binary pump and G1314A variable wavelength detector. Separations were performed with an Eurospher II 100-5 C18 column (150 x 2 mm; Knauer Wissenschaftliche Geräte GmbH Berlin, Germany) with a guard column at 30°C. The mobile phase consisted of acetonitrile 0.1% formic acid (solvent A) and 0.1% formic acid (solvent B). The flow rate was 300 µL min^{-1} and a gradient was used as follows: 95% B (0.0 min), 80% B (0.1 min), 76% B (3.0 min), 60% B (3.1 min), 0% (8.0 min), hold for 2 min and back to 95% B (analysis time 12.0 min). UV detection was performed at 241 nm. Sample injection volume was 2 µl. Aliquots (100 µL) of the supernatant of the reaction mixtures were used directly or diluted with 1 M phosphate buffer and pH was adjusted to 5.0-5.5 with HCl. Standards of TPA, MHET, and BHET were used to generate calibration curves.

Synthesis of MHET

MHET was synthesized as previously described[2] with the following modifications. BHET (28.9 mmol) and KOH (28.1 mmol) were mixed with 60 ml ethylene glycol. Deionized water (120 ml) was added and the mixture was extracted three times with 20 ml chloroform. The aqueous phase was filtered and adjusted to pH 2 with concentrated HCl. The precipitate obtained was washed with hot deionized water. The filtrate was cooled to 4°C and the formed precipitate was collected and dried, resulting in 0.55 g MHET (9% theoretical recovery) with a purity of 97%.

Analysis of PET films by scanning electron microscopy

G-PET films exposed to the enzymes and untreated controls fixed on glass substrates were analyzed by scanning electron microscopy using an SEM (EVO LS10, Carl Zeiss GmbH, Jena, Germany) with a LAB6 cathode (Kimball Physics

Inc., Wilton, NH, USA) and a secondary electron detector. The films were sputter-coated with 25 nm gold prior to imaging (BAL-TEC SCD 050, Leica Biosystems, Nussloch, Germany). Images were captured at an acceleration voltage of 5 kV and a probe current of 3 pA.

Identification of enzymatic hydrolysis products by mass spectrometry

An aliquot (2 µL) of the supernatant from a reaction mixture of PHL7 with amorphous PET films was analyzed by liquid chromatography-high resolution mass spectrometry (LC-HRMS) using an Ultimate 3000 UHPLC System (Thermo Scientific, Waltham, USA) equipped with a SRD-3400 solvent rack, HPG-3200RS binary pump, WPS-3000TRS autosampler, TCC-3000RS column compartment, DAD-3000 detector connected by a Viper fitting system and a Bruker impact II UHR-QqTOF (Ultra-High Resolution Qq-Time-Of-Flight; Bruker Daltonics GmbH Bremen, Germany) mass spectrometer. The software used for data acquisition was Bruker Daltonics Hystar 3.2 SR4 with Compass for otof Series 1.8 (otofControl 4.0.21.1960) and for data analysis Data Analysis 4.2 SR1 (Bruker Daltonics GmbH, Bremen, Germany). Chromatographic separation of the hydrolysis products was performed as described above (section 1.13). MS and AutoMS/MS analyses were carried out in negative MS mode. Parameters of the ESI source: nebulizer gas pressure 3.0 bar (ultra pure N_2), dry gas flow rate 9.0 L min^{-1} (ultra-pure N_2), capillary voltage 2500 V and dry heater temperature 250°C. Mass spectra were detected in the range of *m/z* 100-2000. Quadrupole transfer time was set to 80.0 µs, prepulse storage time to 8.0 µs and the collision cell RF to 1000.0 V. The quadrupole ion energy (MS) was set at -5.0 eV and collision energy (AutoMS/MS) ramped between 0.0 and -60.0 eV for three precursor ions.

Protein crystallization and structure elucidation of PHL7

PHL7 was concentrated to 7.35 mg/mL and crystallized in a vapor diffusion set-up. Initial crystallization conditions were screened as sitting-drop experiments by mixing 100 nL of reservoir solution with an equal amount of protein solution on a Mosquito Xtal 3 pipetting robot (SPT Labtech, Melbourn Hertfordshire, United Kingdom). The crystallization drops were incubated over 100 µL of reservoir solution at 19°C. In total, five different in-house master screens containing 96 different crystallization conditions each were tested. Crystals that grew in 100 mM sodium citrate (pH 5.6), 20% (w/v) PEG 4,000 and 20% (v/v) 2-propanol within 24 h were identified as protein crystals via UV fluorescence photography. Protein crystals for X-ray diffraction experiments were grown at 19°C in hanging drops containing 1 µL of reservoir and 1 µL of protein solution, respectively, over 500 µL of reservoir solution with 2-propanol concentrations between 5 and 20% (v/v). The crystals were plunge-frozen directly from the drop in liquid nitrogen without further cryo-protection. Diffraction data were collected at beamline P14 at the Deutsches Elektronen-Synchrotron (DESY) in Hamburg at a wavelength of 0.97626 Å, processed with XDS[3] and scaled and merged with AIMLESS.[4] The structure was solved by molecular replacement in Phaser-MR[5] using a homology model of PHL-7 generated by SWISS-MODEL[6] as the search model based on the structure of *Thermobifida cellulosilytica* cutinase (PDB: 5LUI).[7] The structure was refined against the diffraction data in phenix.refine[8] and modelled in COOT.[9] Molecular graphics and analyses were prepared in UCSF Chimera.[10]

Analysis of enzymatically produced TPA by ^{1}H-NMR

Purified TPA (2.5 mg) was dissolved in 500 µL D_2O (99.88% D, ARMAR GmbH, Leipzig, Germany) containing 0.15 M NaOH (≥99%, Carl Roth GmbH + Co. KG. Karlsruhe, Germany) The solution was pipetted into a 5-mm NMR tube. ^{1}H solution NMR measurements were performed with a Bruker DRX-600 NB spectrometer equipped with a 5-mm TXI probe at 25°C. The optimized ^{1}H 90° pulse length was 9.0 µs and the recycle delay was 10.0 s. The data were recorded with 128 scans. The phase and the baseline were corrected manually with TopSpin^{TM} 4.0.9 (Bruker). Data processing was performed with MestReNova 12.0.0 (Mestrelab Research S.L., Santiago de Compostela, Spain) and water signals were removed manually. A reference TPA sample (≥98%, Sigma Aldrich, St. Louis, MO, USA) was analyzed using the same conditions.

Analysis of synthesized PET by ^{1}H-NMR

The synthesized PET (5.3 mg) was dissolved in 500 µL hexafluoro-2-propanol (HFIP, ≥99%, Aldrich Chemistry) at room temperature and diluted 16 times in chloroform-*d* ($CDCl_3$, 99.8% D, ARMAR GmbH, Leipzig, Germany) containing 0.003% (v/v) tetramethyl silane (TSM) as an internal standard (δ^H, 0.00 ppm). Then 500 µL solution was pipetted into a 5-mm NMR tube. ^{1}H solution NMR measurements were performed with a Bruker DRX-600 NB spectrometer equipped with a 5-mm TXI probe at 25 °C. The optimized ^{1}H 90° pulse length was 9.0 µs and the recycle delay was 10.0 s. The data were recorded with 1024 scans. The phase and baseline were corrected manually with TopSpin^{TM} 4.0.9 (Bruker). Data processing was performed with MestReNova 12.0.0 (Mestrelab Research S.L., Santiago de Compostela, Spain) and solvent peaks were removed. A reference amorphous PET sample (Goodfellow GmbH, Bad Nauheim, Germany) was analyzed using the same conditions.

The average degree of polymerization (DP) was calculated with the following formula.[11]

M_{PET}=n $M_{PET\ repeating\ unit}$ +62 (1)

It was assumed that each PET chain has two terminal hydroxyl groups.

Analysis of synthesized PET by Fourier-transform infrared spectroscopy (FT-IR)

SUPPORTING INFORMATION

The synthesized PET was analyzed by FT-IR with a Bruker Alpha II (Bruker Optics GmbH Ettlingen) with an attenuated total reflection unit. Measurements were performed in transmission mode (4000 – 400 cm^{-1}) with a resolution of 1 cm^{-1} and 128 scans. A reference G-PET sample was analyzed using the same conditions.

Tables and Figures

Table S1: Location of compost sites, core temperature, moisture content and pH.

Compost Site	Core Temperature [°C]	Moisture Content [%]	pH
Botanical Garden, Leipzig University (2 years old)	3.8 ± 0.2	43.5 ± 0.6	6.3 ± 0.2
South Cemetery, Leipzig (6 months old)	56.0 ± 1.4	67.0 ± 2.1	5.4 ± 0.1
South Cemetery, Leipzig (2 years old)	31.8 ± 1.4	64.4 ± 0.3	6.8 ± 0.1

Table S2: Putative and confirmed hydrolase templates used for the design of the degenerate primer pairs.

Organism	Enzyme	Accession number (NCBI)
Actinoplanes missouriensis 431	lipase	*WP_014444986.1*
Amycolatopsis sp. ATCC 39116	dienelactone hydrolase	WP_020421368
Cellulomonas fimi ATCC 484	alpha/beta hydrolase	*WP_013769543.1*
Deinococcus maricopensis DSM 21211	triacylglycerol lipase	*ADV66860*
Frankia sp. EUN1f	alpha/beta hydrolase	WP_006541329.1
Kineococcus radiotolerans SRS30216	alpha/beta hydrolase	WP_012086425
Kribella flavida DSM 17836	lipase	WP_012918802.1
Micromonospora aurantiaca ATCC 27029	lipase	WP_013286782
Micromonospora aurantiaca ATCC 27029	dienelactone hydrolase	WP_013288056
Micromonospora lupini str. Lupac 08	triacylglycerol lipase	CCH19371.1
Micromonospora sp. L5	lipase	WP_013476469.1
Nocardiopsis dassonvillei subsp. dassonvillei DSM 43111	alpha/beta hydrolase	WP_013155582
Saccharomonospora azurea NA-128	hypothetical protein	WP_005440160.1
Saccharomonospora azurea SZMC 14600	cutinase	EHK89136.1
Saccharomonospora cyanea NA-134	lipase	WP_005458352.1
Saccharomonospora glauca K62	dienelactone hydrolase	WP_005465756.1
Saccharomonospora marina XMU15	dienelactone hydrolase	WP_009155382
Saccharomonospora paurometabolica YIM 90007	lipase	WP_007027147.1
Saccharomonospora viridis DSM 43017	lipase	WP_015787089.1
Saccharomonospora xinjiangensis XJ-54	lipase	WP_006239783.1
Streptomyces albus J1074	dienelactone hydrolase family protein	WP_003948402.1
Streptomyces ambofaciens ATCC 23877	putative secreted lipase	CAJ88461.1
Streptomyces coelicolor A3(2)	lipase	WP_003978149.1
Streptomyces coelicolor A3(2)	lipase	AAD09315.1
Streptomyces davawensis JCM 4913	triacylglycerol lipase	CCK25304.1
Streptomyces flavogriseus ATCC 33331	alpha/beta hydrolase	WP_014156788.1
Streptomyces ghanaensis ATCC 14672	dienelactone hydrolase family protein	WP_004979357.1
Streptomyces griseoflavus Tu4000	triacylglycerol lipase	EFL43114.1
Streptomyces griseus subsp. griseus NBRC 13350	alpha/beta hydrolase	WP_012381325.1
Streptomyces hygroscopicus subsp. jinggangensis 5008	dienelactone hydrolase family protein	WP_014677117.1
Streptomyces pristinaespiralis ATCC 25486	lipase	EFH31823.1
Streptomyces somaliensis DSM 40738	dienelactone hydrolase family protein	WP_010472515.1

Streptomyces sp. S4	dienelactone hydrolase family protein	WP_018894316.1
Streptomyces sp. SirexAA-E	dienelactone hydrolase family protein	WP_014045377.1
Streptomyces sp. SM8	dienelactone hydrolase family protein	WP_008403723.1
Streptomyces venezuelae ATCC 10712	dienelactone hydrolase family protein	WP_015031407.1
Streptomyces zinciresistens K42	lipase	EGX54930.1
Streptosporangium roseum DSM 43021	triacylglycerol lipase	ACZ88272.1
Thermobifida alba	cutinase 1, partial	ADV92525.1
Thermobifida alba	esterase	BAI99230.2
Thermobifida alba	esterase	BAK48590.1
Thermobifida cellulosilytica	cutinase 1, partial	ADV92526.1
Thermobifida cellulosilytica	cutinase 2, partial	ADV92527.1
Thermobifida fusca	cutinase 1, partial	ADV92528.1
Thermobifida fusca	acetylxylan esterase, partial	ADM47605.1
Thermobifida fusca	BTA-hydrolase 2	CAH17554.1
Thermobifida fusca	cutinase, partial	CBY05530.1
Thermobifida fusca	cutinase	CBY05529.1
Thermobifida fusca YX	dienelactone hydrolase family protein	WP_011291330.1
Thermobifida fusca YX	triacylglycerol lipase	AAZ54920.1
Thermobifida halotolerans	serine hydrolase	AFA45122.1
***Thermomonospora curvata* DSM 43183**	lipase	WP_012850775.1
***Thermomonospora curvata* DSM 43183**	triacylglycerol lipase	WP_012851645.1
Uncultured bacterium	cutinase-like protein	AEV21261.1

Table S3: Number of clones forming clearing zones on agar plates containing tributyrin, PCL or PET nanoparticles. The clones were obtained by cloning of the PCR products amplified with degenerate primers.

**Composts**	**Number of forming clearing zones on agar plates containing**		
	**Tributyrin**	**PCL**	**PET**
Botanical garden, Leipzig University (2 years old)	43	15	5
South cemetery Leipzig (6 months old)	63	22	11
South cemetery Leipzig (2 years old)	30	22	8

Table S4: Polyester hydrolases (PHL) isolated from three composts. Sequences are deposited at the European Nucleotide Archive (ENA).

	ENA Accession Number	Compost	Amino acid sequence identity in % with hydrolases from different genera of actinobacteria (98-99% sequence coverage)
PHL1	LT571440	Botanical Garden, Leipzig University	99.2 % *Actinomadura hallensis*
PHL2	LT571441	Botanical Garden, Leipzig University	91.5 % *Actinomadura hallensis*
PHL3	LT571442	South cemetery Leipzig	74.3 % *Actinoalloteichus fjordicus*<br>73.6 % *Streptomyces albulus*<br>72.8 % *Actinokineospora fastidiosa*<br>72.7 % *Micromonospora humi*
PHL4	LT571443	South cemetery Leipzig	65.6 % *Streptomyces sclerotialus*<br>65.3 % *Amycolatopsis roodepoortensis*<br>64.9 % *Actinomadura fibros*<br>64.6 % *Actinoplanes atraurantiacus*
PHL5	LT571444	South cemetery Leipzig	66.9 % *Streptomyces finlayi*<br>66.7 % *Amycolatopsis orientalis*<br>66.7 % *Actinokineospora spheciospongiae*<br>66.3 % *Actinomadura fibrosa*
PHL6	LT571445	South cemetery Leipzig	66.3 % *Streptomyces sclerotialus*<br>65.9 % *Amycolatopsis orientalis*<br>65.5 % *Actinomadura fibrosa*<br>65.1 % *Actinoplanes atraurantiacus*
PHL7	LT571446	South cemetery Leipzig	74.6 % *Actinoalloteichus fjordicus*<br>73.2 % *Micromonospora humi*<br>73.2 % *Streptomyces albulus*<br>72.7 % *Actinokineospora fastidiosa*

Table S5: Data collection and refinement statistics of the crystal structure of PHL7

**Data collection**	
Resolution range [Å]	49.22 - 1.30 (1.346 - 1.30)[a]
Space group	$P2_12_12_1$
Wavelength [Å]	0.9763
Temperature [K]	100
Unit cell dimensions a, b, c [Å]	56.35, 97.68, 101.10
Reflections	1,548,209 (58,550)
Unique reflections	127,602 (8,194)
Multiplicity	12.1 (7.1)
Completeness (%)	92.80 (60.10)[b]
I/σ(I)	19.95 (1.39)
R_{merge}	0.06961 (1.199)
R_{pim}	0.02018 (0.4746)
$CC_{1/2}$	1.000 (0.587)
Wilson B-factor	14.06
**Refinement**	
Reflections used in refinement	127,577 (8,191)
Reflections used for R_{free}	6,202 (440)
R_{work}	0.1316 (0.2499)
R_{free}	0.1593 (0.2805)
No. of non-hydrogen atoms:	4,932
Protein	4,078
Solvent	852
Na^+	2
RMS deviations	
Bonds [Å]	0.006
Angles [°]	0.94
Ramachandran	
favored	98.05 %
allowed	1.95 %
outliers	0.00 %
Clashscore	1.73
Average B-factor	19.55
protein	16.23
solvent	35.41
Na^+	18.90
PDB ID	7NEI

[a] Values for highest resolution bin in parentheses.

[b] Completeness drops at high resolution due to the rectangular shape of the detector. Completeness remains above 92.9 % up to a resolution of 1.42 Å.

Table S6: Number of enzyme-ligand complexes in each cluster obtained from a hierarchical clustering distance matrix. Each cluster represents a distinct binding mode.

	Cluster		
Enzyme-ligand complex	1	2	3
PHL7-EMT	31	29	40
PHL7-MHET	50	30	20
PHL7-TPA	73	24	3
LCC-EMT	33	19	48
LCC-MHET	75	16	9
LCC-TPA	90	6	4

Table S7: Mass spectrometric data of hydrolysis products detected in the supernatant of reaction mixtures of PHL7 with amorphous PET films. The retention time, ionic species and error of the calculated *m/z* are indicated. The accurate mass of the unidentified products U1 and U2 was calculated using the sum formula with the lowest error.

	Retention time	Ionic species	Observed *m/z*	Calculated *m/z*	Error (ppm)
TPA	3.05-3.26	$(M-H)^-$	165.0184	165.0193	-5.5
		$(M+H_2PO_4)^-$	262.9956	262.9962	-2.3
		$(2M-H)^-$	331.0454	331.0459	-1.5
		$(2M-2H+Na)^-$	353.0274	353.0279	-1.4
		$(2M-2H+K)^-$	369.0012	369.0018	-1.6
MHET	3.69-3.90	$(M-H)^-$	209.0450	209.0455	-2.4
		$(M+H_2PO_4)^-$	307.0219	307.0224	-1.6
		$(2M-H)^-$	419.0977	419.0984	-1.7
		$(2M-2H+Na)^-$	441.0801	441.0803	-0.5
		$(2M-2H+K)^-$	457.0538	457.0543	-1.1
BHET	4.25-4.46	$(M-H)^-$	253.0714	253.0718	-1.6
		$(M+Cl)^-$	289.0483	289.0484	-0.3
		$(M-H+Na+Cl)^-$	311.0306	311.0304	0.6
		$(M-H+K+Cl)^-$	327.0043	327.0043	0.0
		$(M+H_2PO_4)^-$	351.0485	351.0487	-0.5
U1	5.30-5.52	$(M-H)^-$	251.0922	251.0925	-1.2
		$(M-H+Na+Cl)^-$	309.0507	309.0511	-1.3
		$(M-H+K+Cl)^-$	325.0250	325.0251	-0.3
		$(M+H_2PO_4)^-$	349.0693	349.0694	-0.3
EMT	5.59	$(M-H)^-$	401.0877	401.0878	-0.2
		$(M-2H+Na)^-$	423.0712	423.0698	3.3
		$(M-H+Na+Cl)^-$	459.0480	459.0464	3.5
		$(M+H_2PO_4)^-$	499.0638	499.0647	-1.8
EBT	5.66-5.80	$(M-H)^-$	357.0609	357.0616	-2.0
		$(M-2H+Na)^-$	379.0433	379.0435	-0.5
		$(M-2H+K)^-$	395.0175	395.0175	0.0
		$(M-H+K+Cl)^-$	430.9941	430.9942	-0.2
		$(M+H_2PO_4)^-$	455.0381	455.0385	-0.9
U2	6.36-6.57	$(M-H)^-$	399.1078	399.1085	-1.8
		$(M-2H+Na)^-$	421.0900	421.0905	-1.2
		$(M-H+Na+Cl)^-$	457.0664	457.0672	-1.8
		$(M+H_2PO_4)^-$	497.0844	497.0854	-2.0
		$(2M-H)^-$	799.2212	799.2244	-4.0

Table S8: Mass spectrometric data of hydrolysis products detected in the supernatant of reaction mixtures of PHL7 with amorphous PET films. All products showed the $(C_7H_5O_2)^-$ fragment with the calculated *m/z* 121.0295 similar to TPA. BHET, MHET and the unidentified product U1 contain 1 TPA moiety. 1,2-ethylene-mono-terephthalate-mono(2-hydroxyethyl terephthalate) (EMT), 1,2-ethylene-bis-terephthalate (EBT) and the unidentified product U2 showed in addition fragments with an *m/z* of 181.0659 $(C_{13}H_9O_1)^-$ and *m/z* 225.0557 $(C_{14}H_9O_3)^-$ corresponding to 2 TPA moieties.

	Detected fragment *m/z*	Best possible molecular formula	Calculated *m/z*	Error (ppm)
TPA	121.0295; 121.0292; 121.0291	$(C_7H_5O_2)^-$	121.0295	0.0 - 3.3
MHET	121.0293	$(C_7H_5O_2)^-$	121.0295	1.7
	165.0555; 165.0549; 165.0548	$(C_9H_9O_3)^-$	165.0557	1.2 - 5.5
BHET	121.0293; 121.0290	$(C_7H_5O_2)^-$	121.0295	1.7 - 4.1
U1	121.0293; 121.0295	$(C_7H_5O_2)^-$	121.0295	0.0 - 1.7
	207.1031; 207.1020	$(C_{12}H_{15}O_3)^-$	207.1027	1.9 - 3.4
EMT	121.0293; 121.0294; 121.0290	$(C_7H_5O_2)^-$	121.0295	0.8 - 4.1
	181.0655; 181,0651	$(C_{13}H_9O_1)^-$	181.0659	2.2 - 4.4
	199.0762	$(C_{13}H_{11}O_2)^-$	199.0765	1.5
	225.0556; 225.0554	$(C_{14}H_9O_3)^-$	225.0557	0.4 - 1.3
EBT	121.0293; 121.0294; 121.0289	$(C_7H_5O_2)^-$	121.0295	0.8 - 5.0
	181.0653; 181.0664	$(C_{13}H_9O_1)^-$	181.0659	2.8 - 3.3
	225.0557; 225.0561	$(C_{14}H_9O_3)^-$	225.0557	0.0 - 1.8
	335.0534; 335.0535	$(C_{17}H_{12}O_6Na)^-$	335.0537	0.6 - 0.9
U2	121.0292; 121.0293; 121.0292	$(C_7H_5O_2)^-$	121.0295	1.7 - 2.5
	181.0651; 181.0655; 181.0666	$(C_{13}H_9O_1)^-$	181.0659	3.9 - 4.4
	225.0553; 225.0554; 225.0556	$(C_{14}H_9O_3)^-$	225.0557	0.4 - 1.8
	355.1183; 355.1187	$(C_{20}H_{19}O_6)^-$	355.1187	0.0 - 1.1

Table S9: Manufacturer and crystallinity of the thermoform PET containers.

Manufacturer	Crystallinity (%)
TYPACK (Santiago, Chile)	3.1
Versapak (Kent, United Kingdom)	1.4
Guillin (Ornans, France)	4.5
Mercato SP ZOO (Olsztyn, Poland)	3.8

Table S10: Objectives used for VSI

**Objective** (magnification / numerical aperture)	**Field of view**	**Virtual pixel size**	**Measurement mode**
Nikon DI (20× / 0.40)	877.20 µm × 660.48 µm	0.65 µm × 0.65 µm	VSI
Nikon DI (100× / 0.70)	175.44 µm × 132.10 µm	0.13 µm × 0.13 µm	VSI
Nikon EPI (150× / 0.90)	116.96 µm × 88.06 µm	0.09 µm × 0.09 µm	confocal

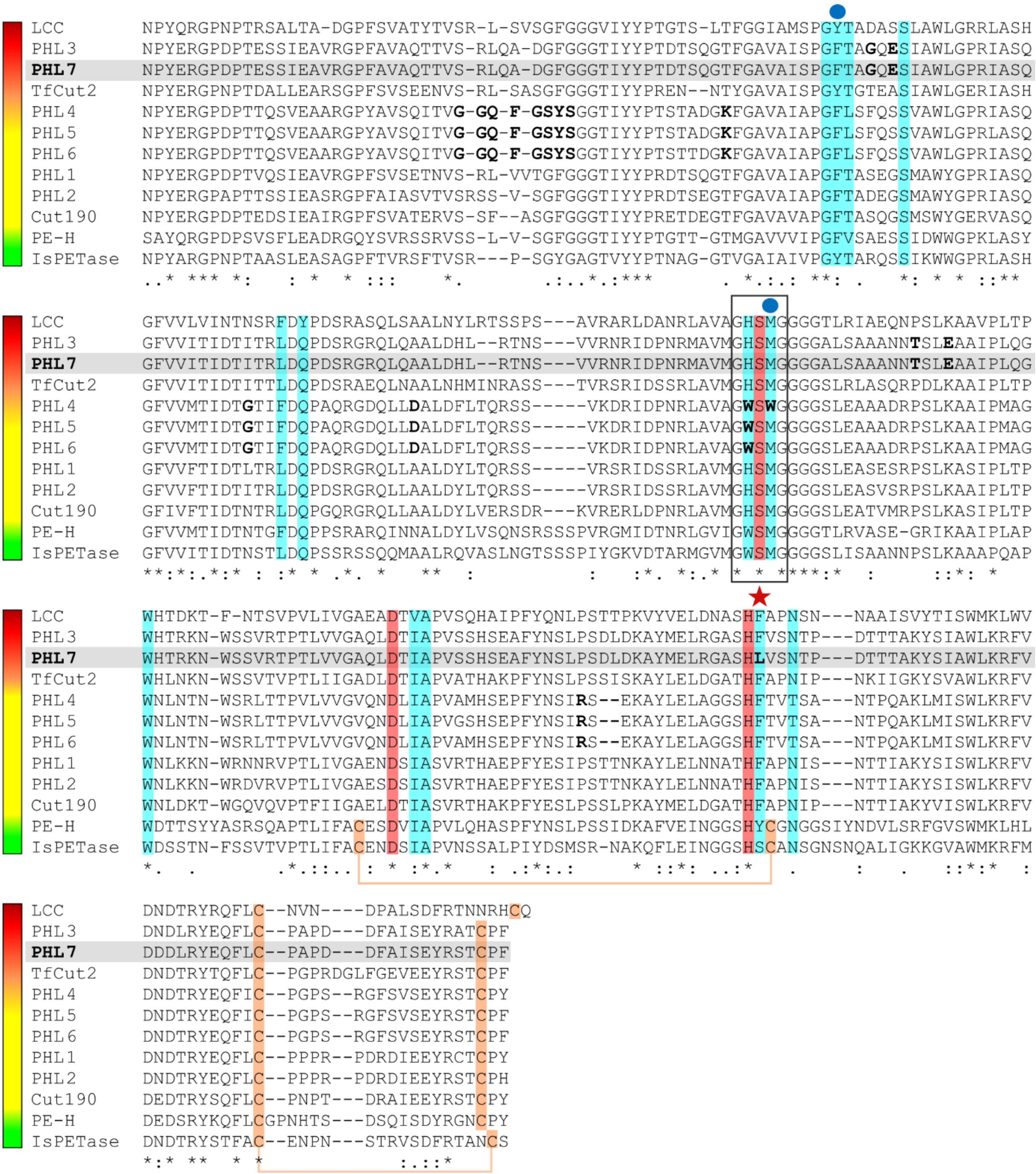

Figure S1: Multiple protein sequence alignment of selected polyester hydrolases. The sequences were ranked according to the thermostability of the protein. Active site residues are highlighted in cyan and the catalytic triad is highlighted in red. Cysteine residues forming disulfide bridges are highlighted in orange. The oxyanion hole is marked with blue dots. The residue 210 (PHL7 numbering) is marked with a red star. The GXSXG box is marked in bold. Uncommon residues found in the PHL are also marked in bold. The N-terminus was trimmed. The sequences can be retrieved from the NCBI server with the accession numbers: G9BY57 (LCC), BAO42836 (Cut190), CBY05530 (TfCut2), WP_088276085 (PE-H), A0A0K8P6T7 (*Is*PETase). The alignment was created with T-Coffee Expresso.[12]

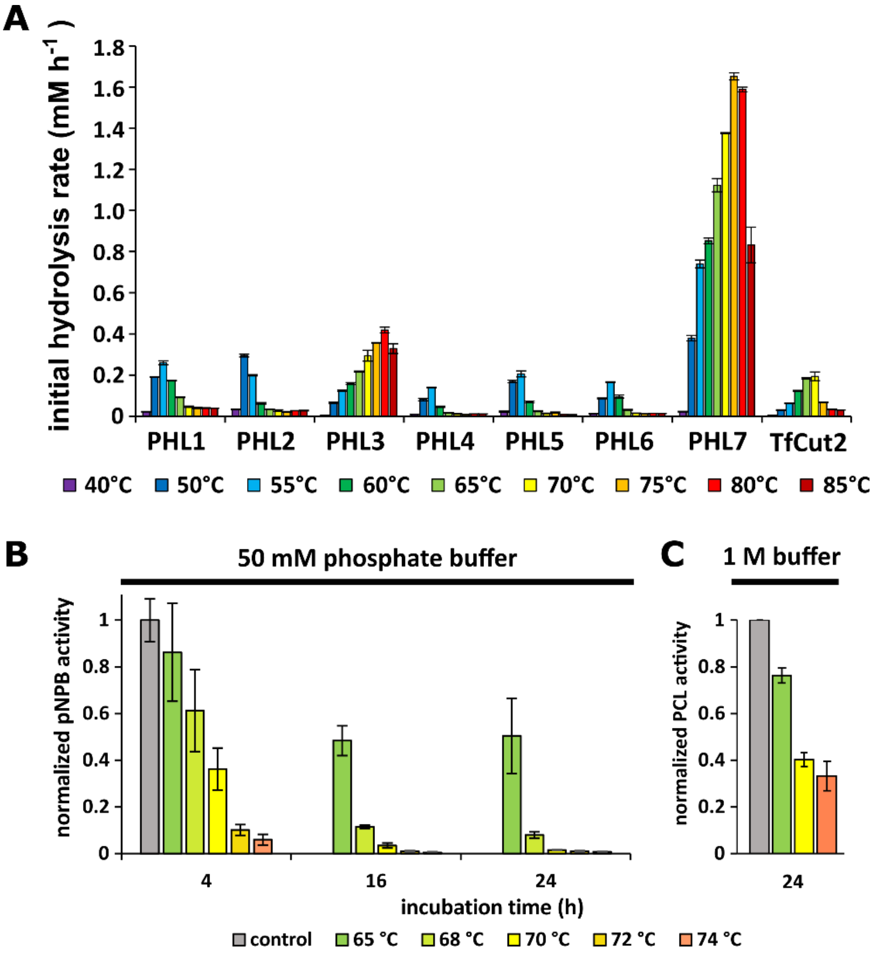

Figure S2: (A) Initial hydrolysis rates of PHL (sum of TPA, MHET and BHET released from G-PET films within 1 h of reaction) at different temperatures. (B) Thermal stability of PHL7. Residual activity of PHL7 was determined with pNPB as substrate following incubations in 50 mM phosphate buffer for 4, 16, and 24 h at temperatures between 65°C and 74°C. (C) Residual activity with PCL as substrate was determined following incubations in 1 M phosphate buffer for 24 h at temperatures between 65°C and 74°C.

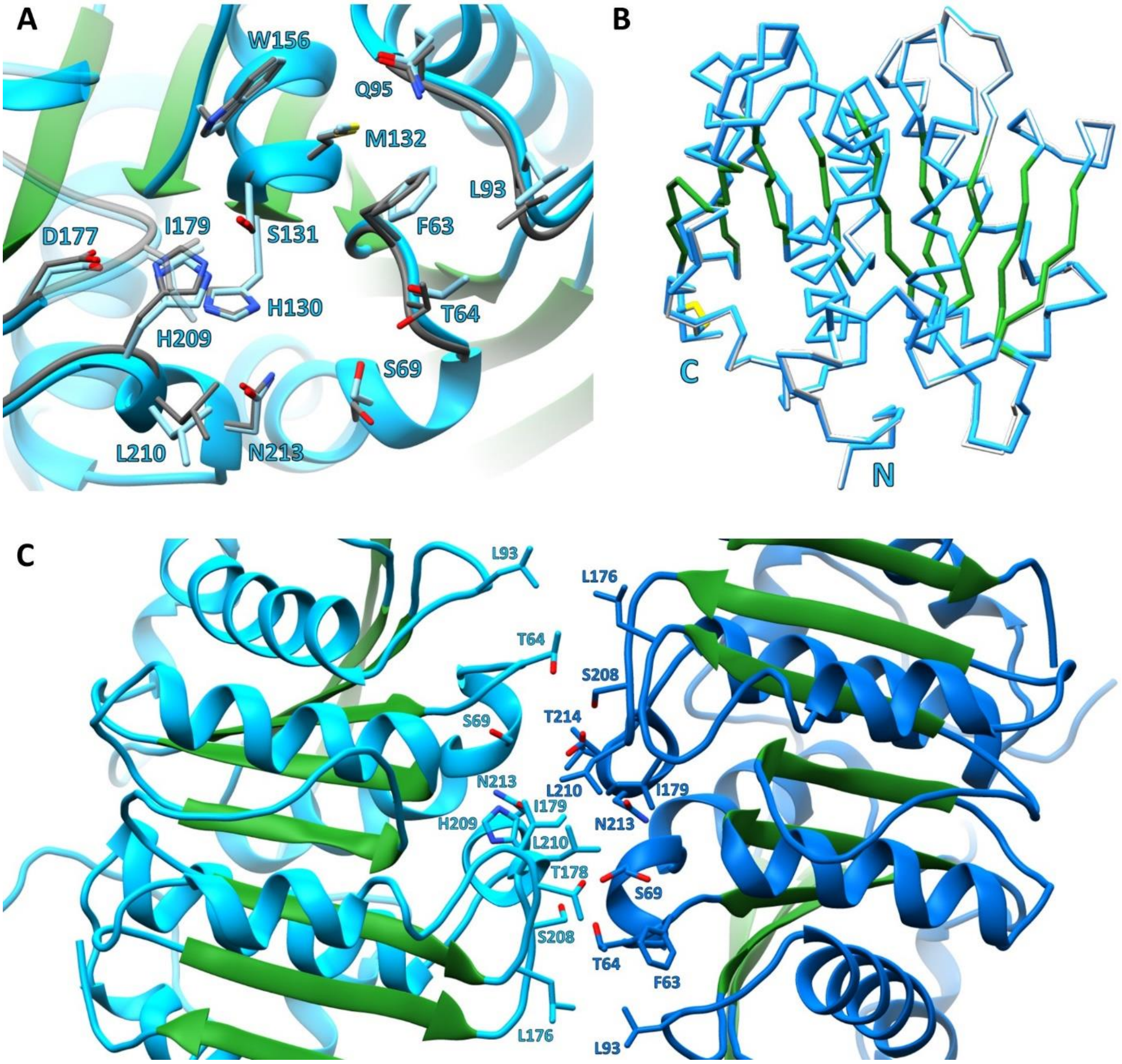

Figure S3: Comparison of the two chains in the asymmetric unit of the PHL7 crystals. (A) Structural alignment of the active site residues of chain A (light blue, strands in green) and chain B (grey) in the asymmetric unit of the PHL7 crystals. Most amino acids align well except for deviations at F63, T64, S69, L93 and L210. These residues are involved in crystal contacts between chain A and B. (B) Structural alignment of the C_α trace of PHL7 chain A (light blue and green) and chain B (light grey). Both chains are almost identical indicated by an RMSD of 0.3 Å (alignment of all 258 C_α atoms). The disulfide bridge between C242-C257 is depicted as yellow sticks and located close to the C-terminus. (C) Interface between chains A (light blue, strands in green) and B (dark blue, strands in green). The two molecules interact mainly via active site residues including F63, T64, S69, L93, I179, H209, L210 and N213 and via the other residues L176, T178, S208 and T214.

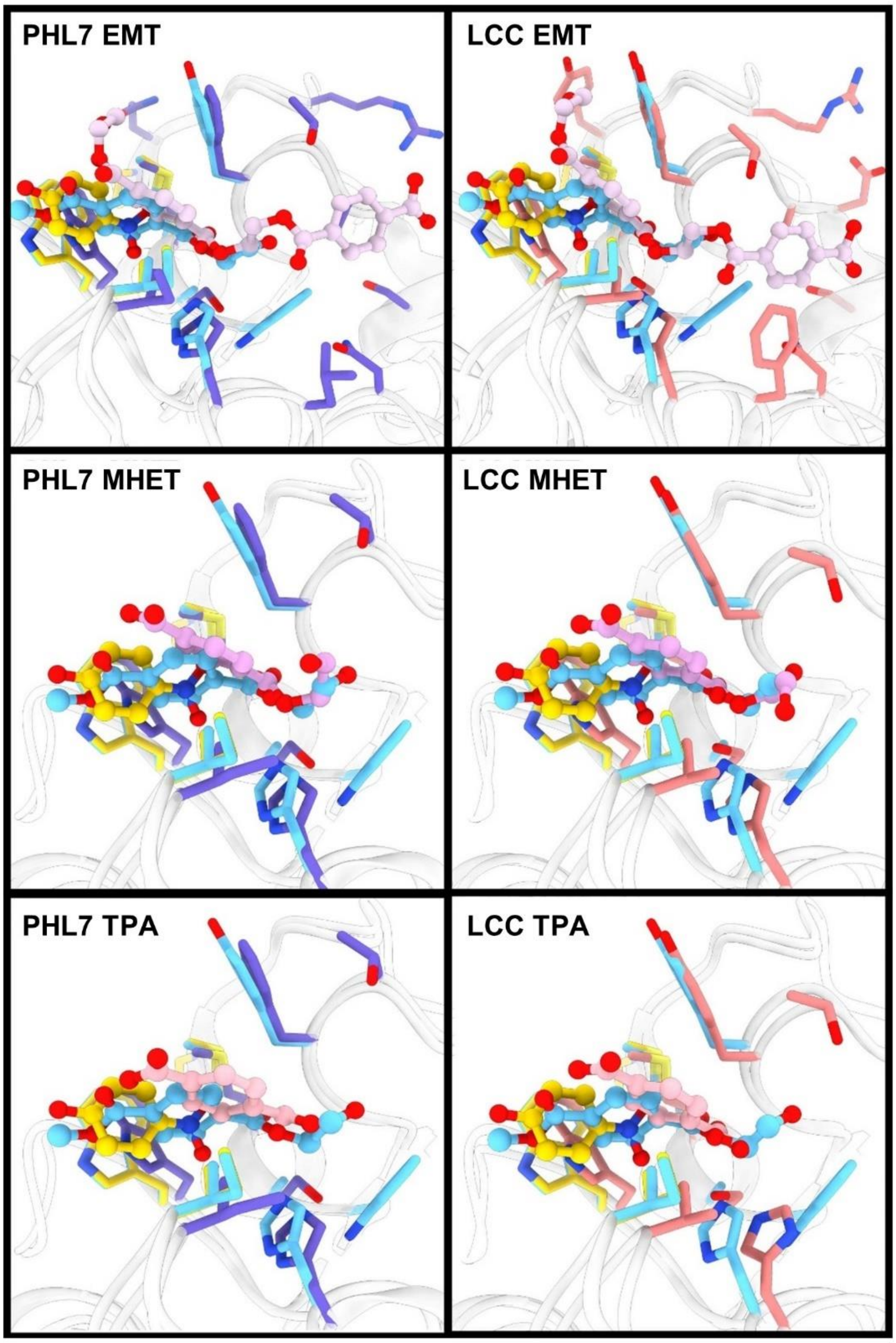

Figure S4: Superimposition of a representative structure of EMT, MHET and TPA clusters with the solved structure of *Is*PETase in complex with HEMT and *p*-nitrophenol for PHL7 and LCC. The lowest binding energy EMT, MHET and TPA docking poses (balls and sticks, pink) positioned a terephthalic ring in the same groove as seen for *p*-nitrophenol (balls and sticks, yellow) and HEMT (balls and sticks, cyan) in solved structures of *Is*PETase. Crystal structures of *Is*PETase are represented in sticks in yellow and cyan, PHL7 is represented in sticks in dark blue and LCC is represented in sticks in salmon.

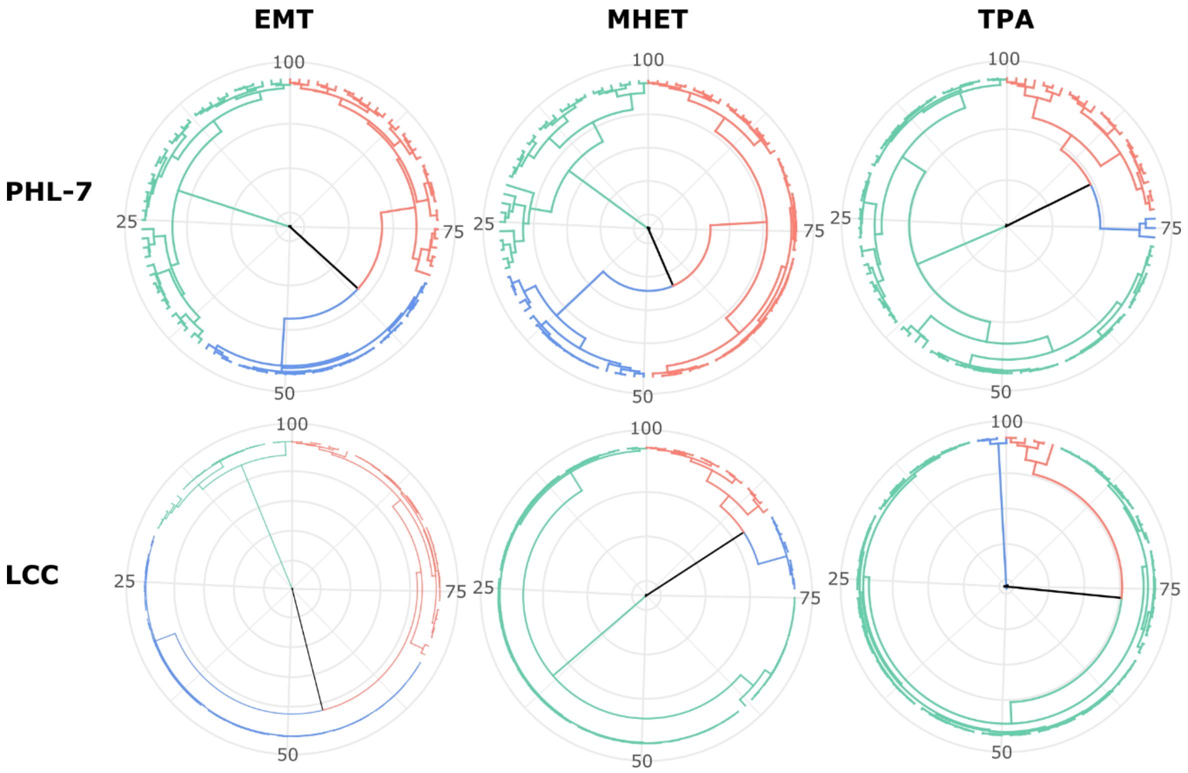

Figure S5: Radial dendrogram of the hierarchical clustering distance matrix of the 100 lowest binding energy complexes of LCC and PHL7 and their corresponding ligands (EMT, MHET and TPA). Cluster 1 is marked in green, cluster 2 in red and cluster 3 in blue. Each cluster represents a distinct binding mode. Cluster abundances are indicated in Table S6.

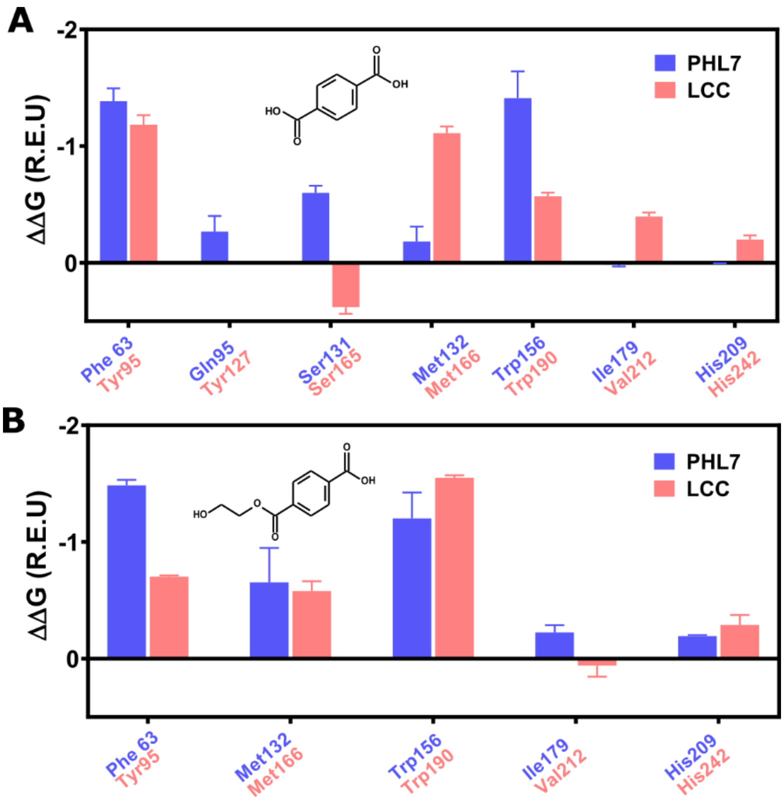

Figure S6: Predicted per-residue binding energy contributions for TPA (A) and MHET (B) in PHL7 and LCC. The best five complexes from a total of 40,000 with the lowest interface binding energy and a RMSD lower than 1.5 Å in relation to the HEMT crystal structure[13] were selected.

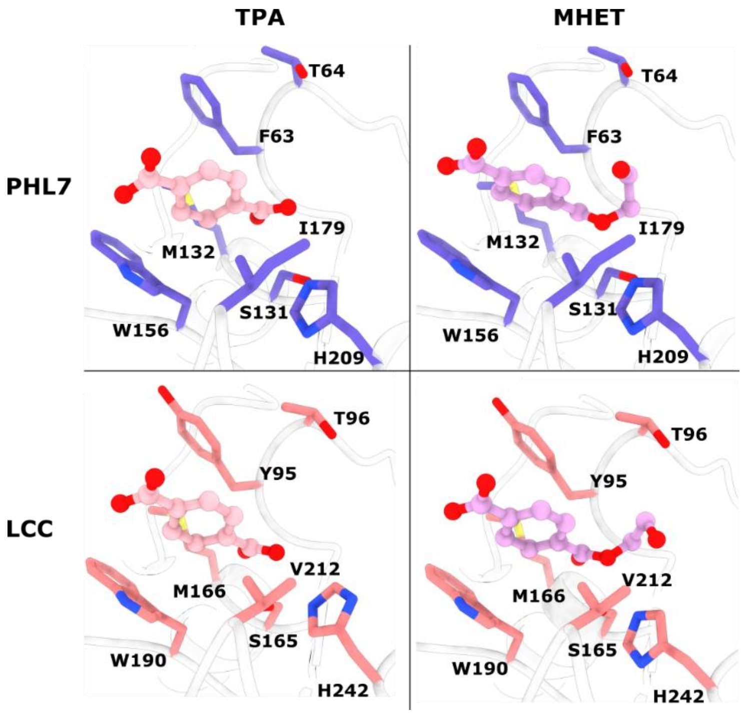

Figure S7: Lowest RMSD pose of the 0.25% best interface binding energy complexes. Top: PHL7 in complex with TPA and MHET. Bottom: LCC in complex with TPA and MHET.

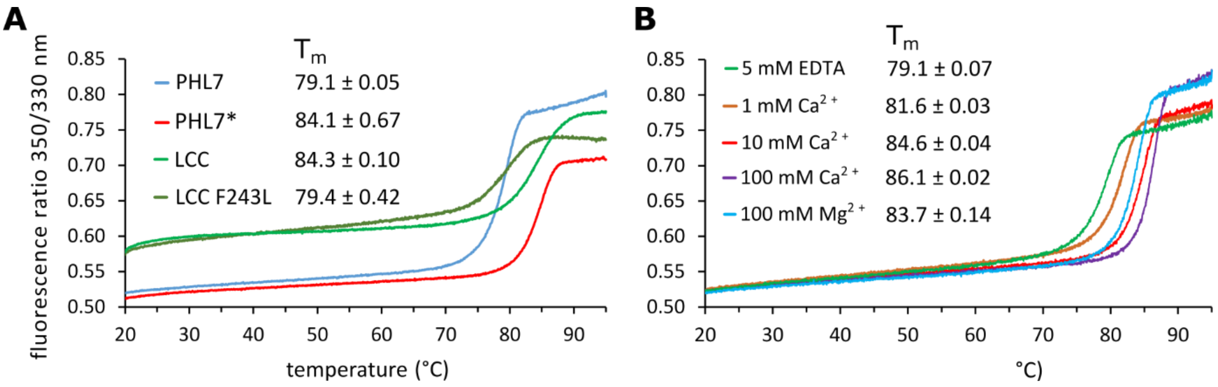

Figure S8: Thermostability of PHL7, LCC, and the LCC variant F243L determined by nano differential scanning fluorimetry. A) Melting curves and melting points of PHL7, LCC, and LCC F243L in 50 mM phosphate buffer. The melting point of PHL7 in 1 M phosphate buffer is marked (*). B) Melting curves and melting points of PHL7 in 10 mM Tris, 1 mM NaCl buffer, pH7.4 with EDTA, $CaCl_2$ and $MgCl_2$. T_m values are shown as mean values ± SD, n=3.

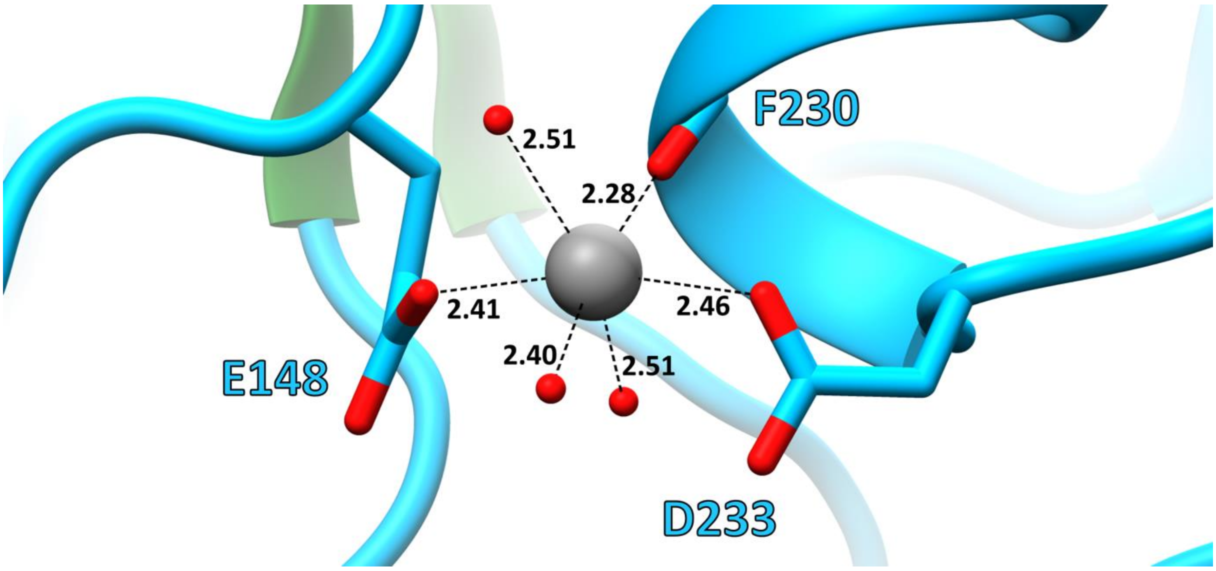

Figure S9: Sodium binding site at ~27 Å distance to the PHL7 active site. The metal ion (grey) is complexed by the carboxyl side chains of E148 and D233, the backbone carbonyl oxygen of F230 and three water molecules. The binding sites in chains A and B are identical. All distances in Å.

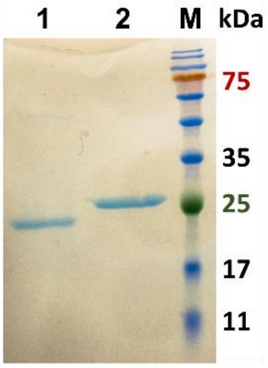

Figure S10: SDS-PAGE of homogeneous LCC (lane 1) and PHL7 (lane 2) preparations. M: pre-stained molecular weight marker, 11-180 kDa.

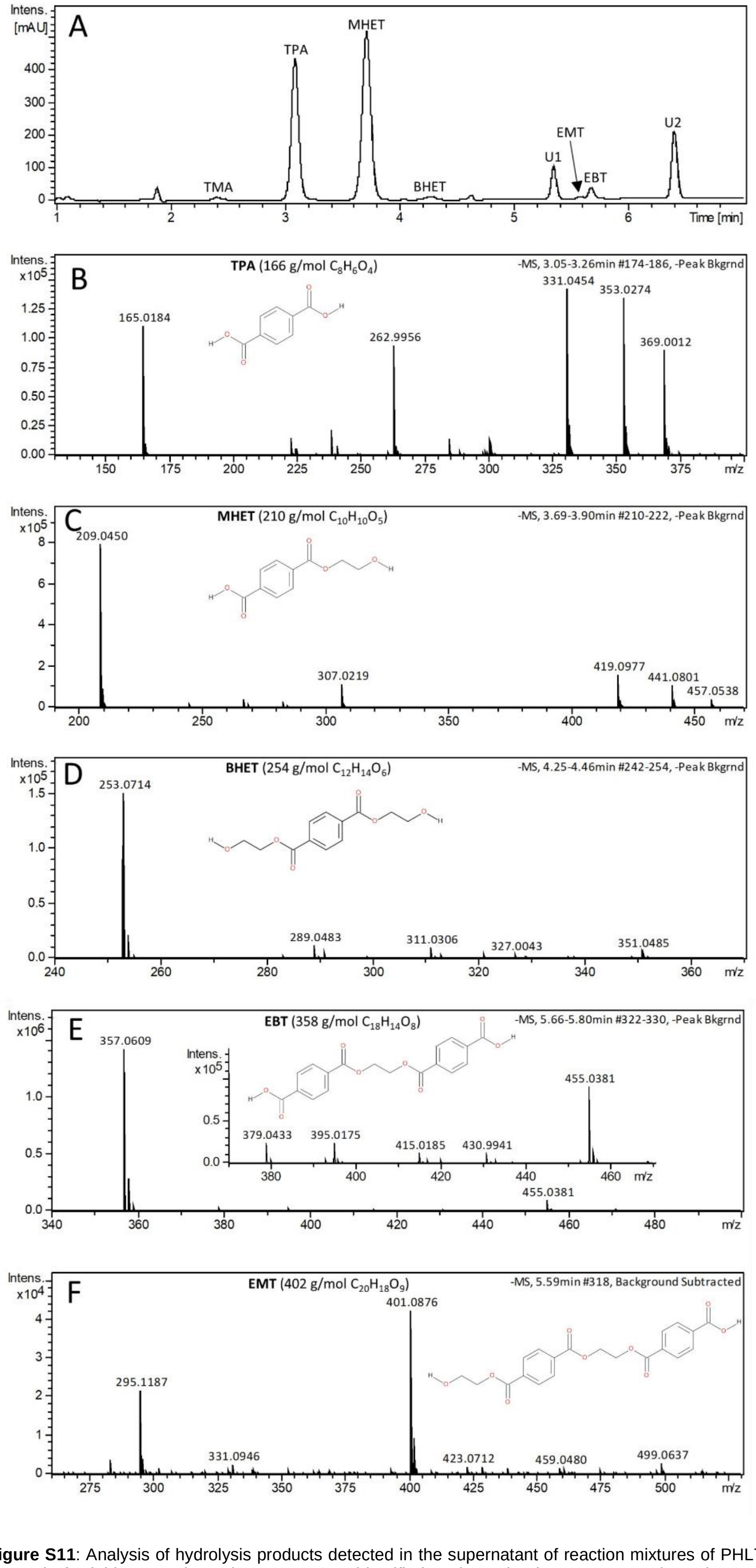

Figure S11: Analysis of hydrolysis products detected in the supernatant of reaction mixtures of PHL7 with G-PET films. (A) HPLC (detection at 254 nm) of soluble aromatic products. U1, U2: unidentified products. (B-F): Mass spectra (negative mode). (B) TPA, (C) MHET, (D) BHET, (E) 1,2-ethylene-mono-terephthalate-mono(2-hydroxyethyl terephthalate) (EMT), (F) 1,2-ethylene-bis-terephthalate (EBT). Trimesic acid (TMA): internal standard.

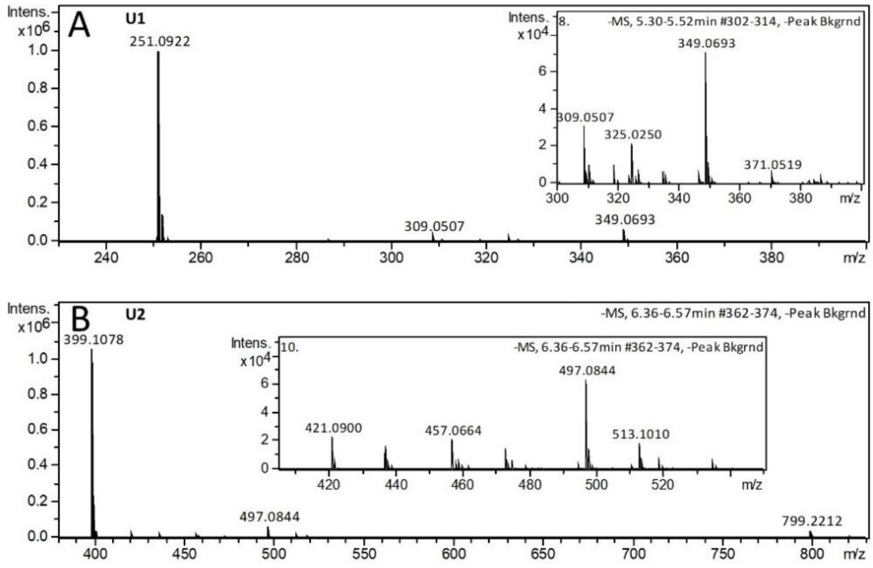

Figure S12: MS spectra (negative mode) of the unidentified hydrolysis products U1 and U2 with molecular weights of (A) 252 g/mol and (B) 400 g/mol, respectively. U1 (*m/z* = 251.0922; calculated formula $C_{13}H_{16}O_5$; -1.2 ppm mass error) differs from the *m/z* of BHET by 2u; U2 (*m/z* = 399.1078; calculated formula $C_{21}H_{20}O_8$; -1.8 ppm mass error) differs from the *m/z* of EMT by 2u. Calculated molecular formulas for U1 and U2 could be explained by a replacement of an oxygen by CH_2 in BHET and EMT, respectively.

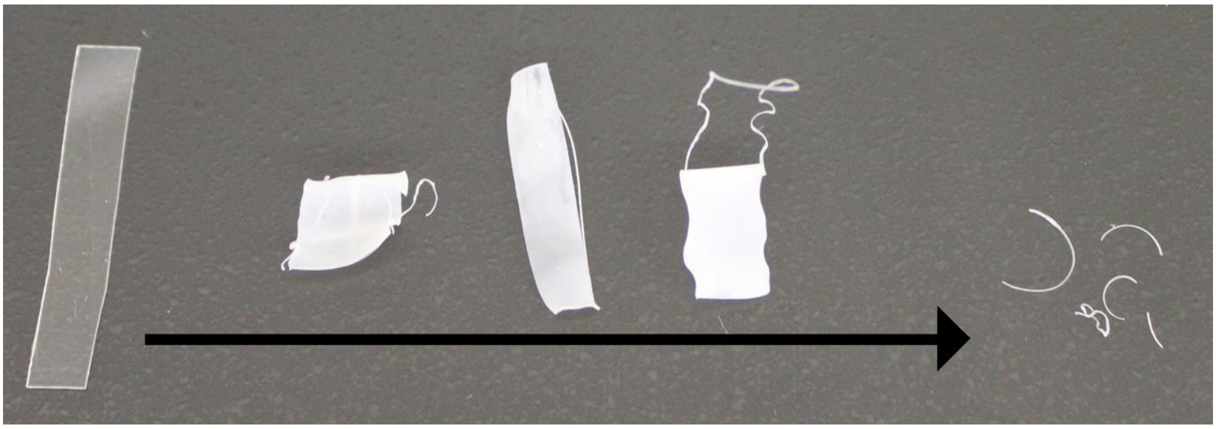

Figure S13: Stages of degradation of a G-PET film by PHL7 showing only cutting edges of the film remaining after a reaction time of 24 h. The edges (approximately 0.5% of the initial film weight) originated from cutting the film with a scissor prior to enzymatic hydrolysis.

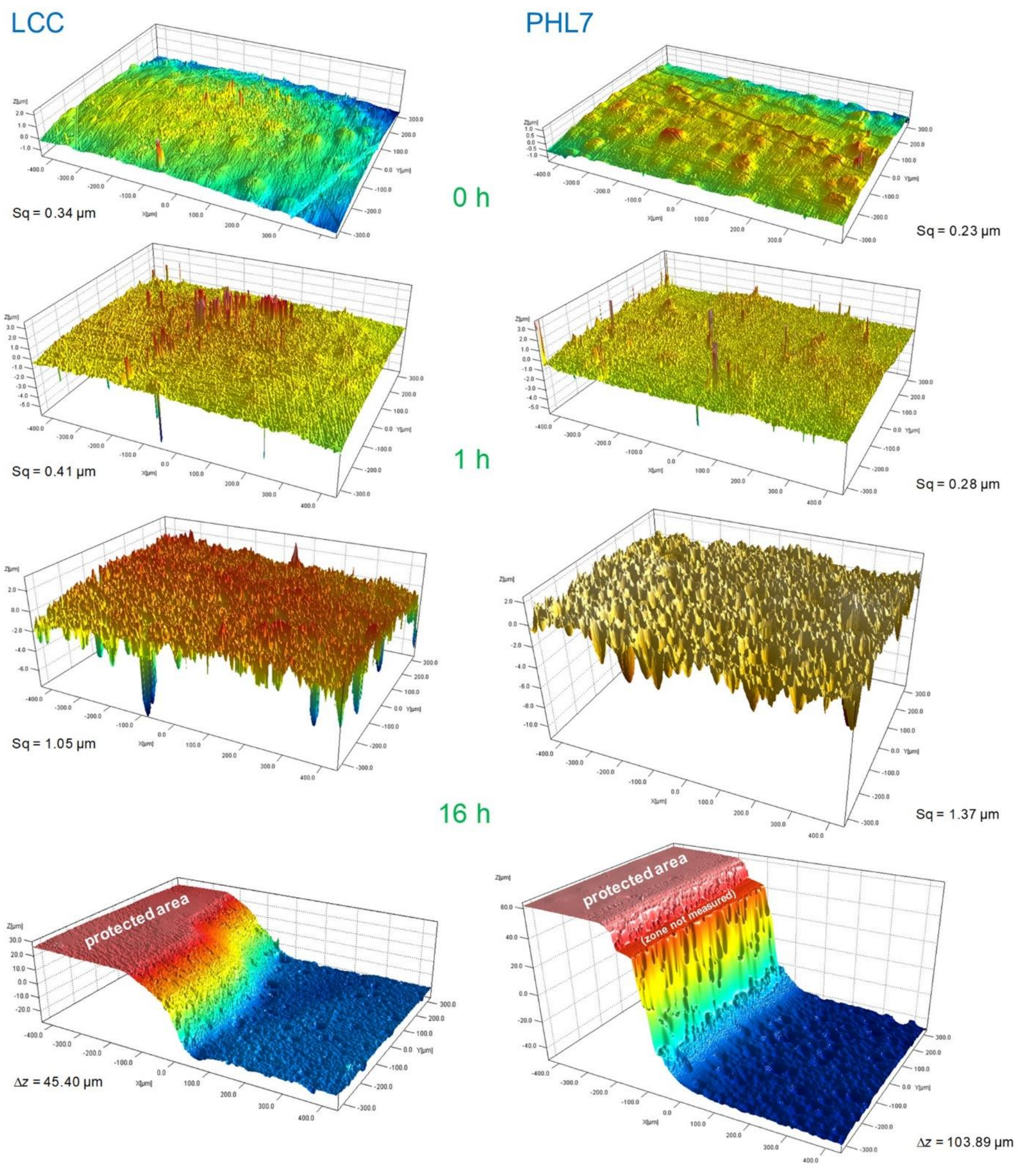

Figure S14: Surface topographies obtained by VSI of G-PET films after different exposure times with LCC and PHL7. The upper images show a progressive increase in roughness, indicated by Sq values representing the standard deviation of height. The measurements for 0 h and 1 h, performed at the same respective positions, were used for the calculation of rate maps and rate spectra (Figure 5). The lower images show the degradation edges after an exposure time of 16 h, with Δz values referring to the most frequent heights. The protected area was masked with Teflon tape as control.

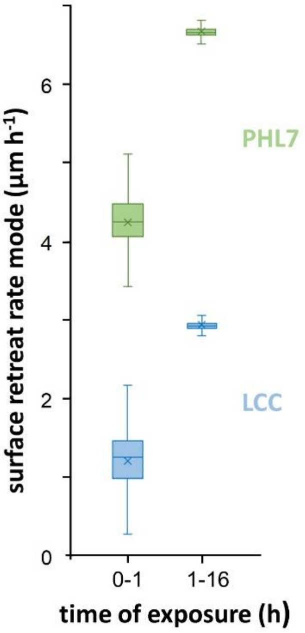

Figure S15: Degradation rates for two exposure periods, measured by VSI. The rates are averaged for the periods 0 h - 1 h and 1 h - 16 h, respectively. The box plots show their variations across the surface area with the horizontal lines indicating the 25%, 50% and 75% percentiles and the crosses indicating the arithmetic mean values. The surface-normal retreat is based on the most frequent heights, taken from the step images shown in Figure 4 and Figure S14.

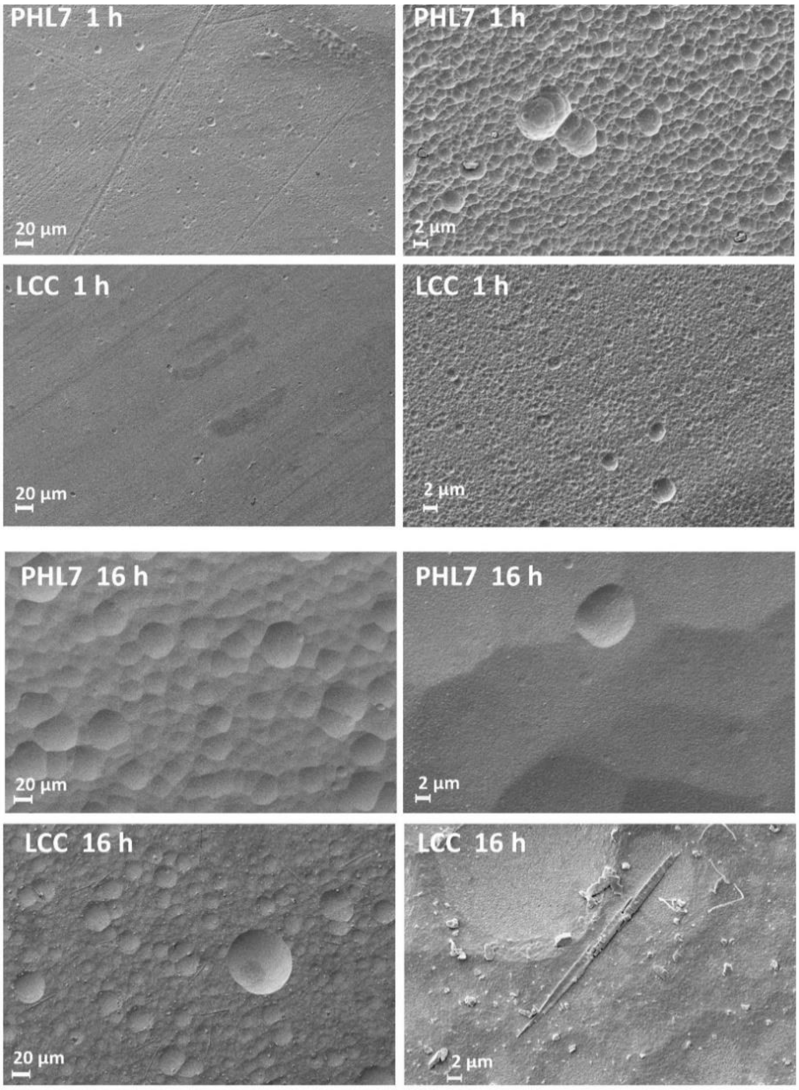

Figure S16: Scanning electron microscopic analysis of amorphous G-PET films following exposure with PHL7 and LCC for 1 h and 16 h.

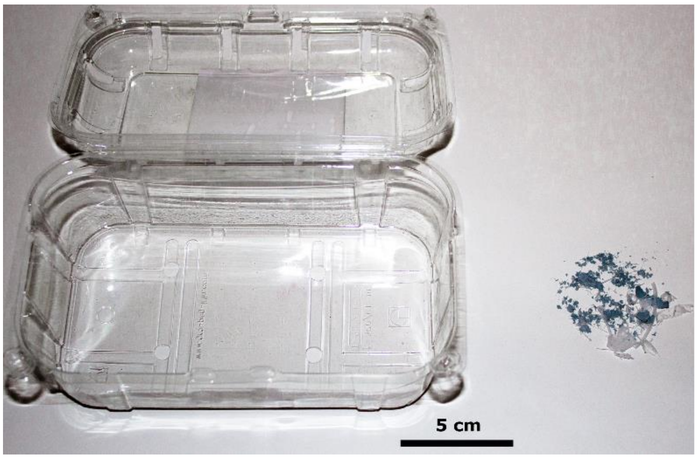

Figure S17: PET clamshell container before and after degradation with PHL7.

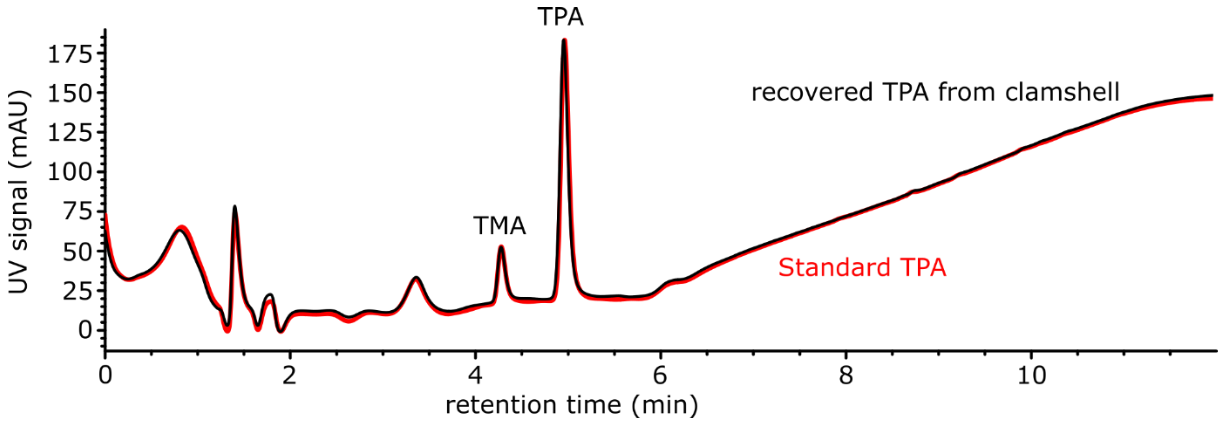

Figure S18: HPLC chromatogram of purified TPA recovered from the enzymatic hydrolysate of a PET clamshell container degraded with PHL7. Black: Recovered TPA. Red: TPA standard (98% purity). TMA: trimesic acid, internal standard.

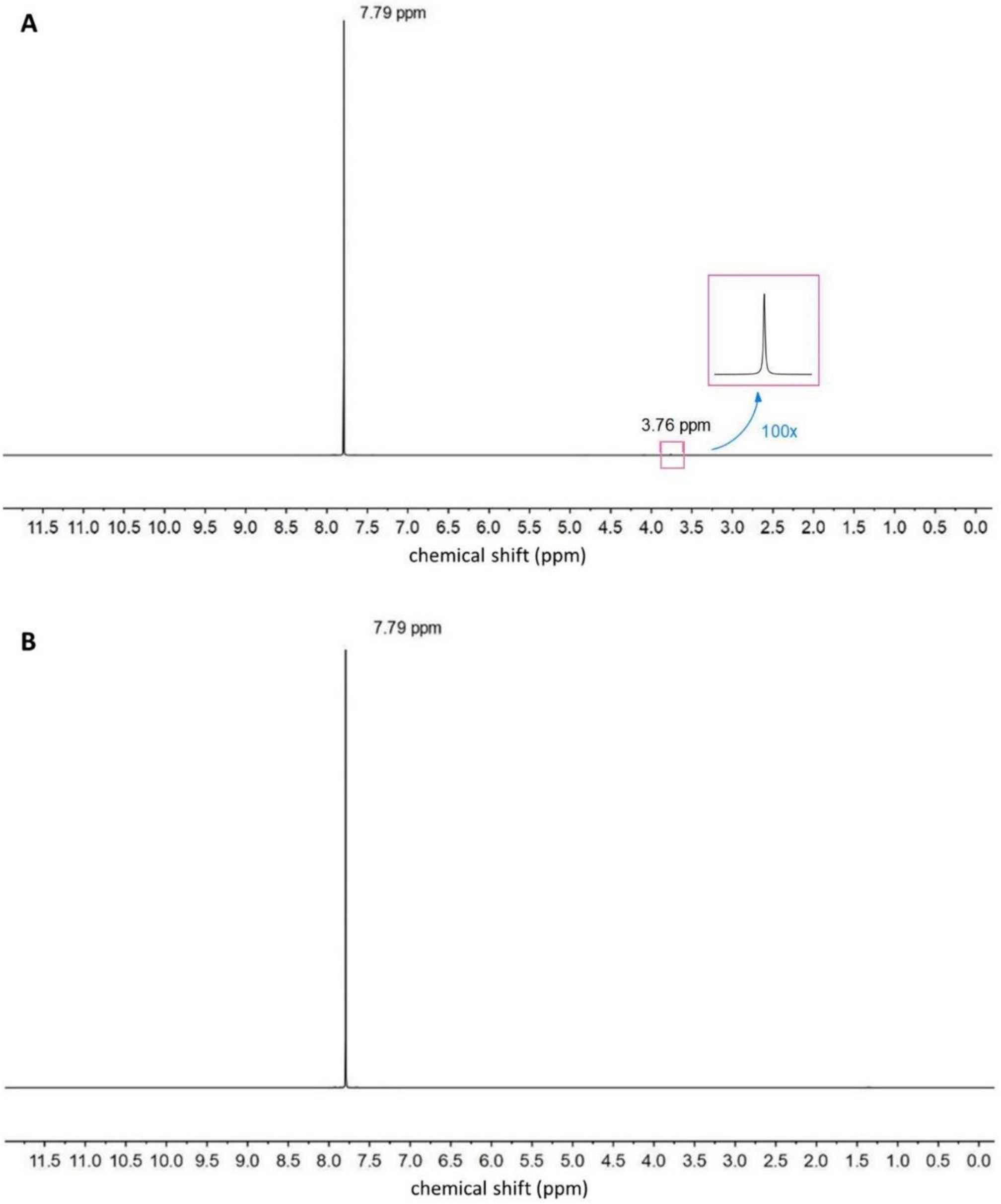

Figure S19: ^{1}H NMR spectra of purified TPA recovered from the enzymatic hydrolysate of a PET clamshell container degraded with PHL7 (A) and a TPA standard (98% purity) (B). The peak at 7.79 ppm corresponds to the hydrogen nuclei of the benzene ring.[14]

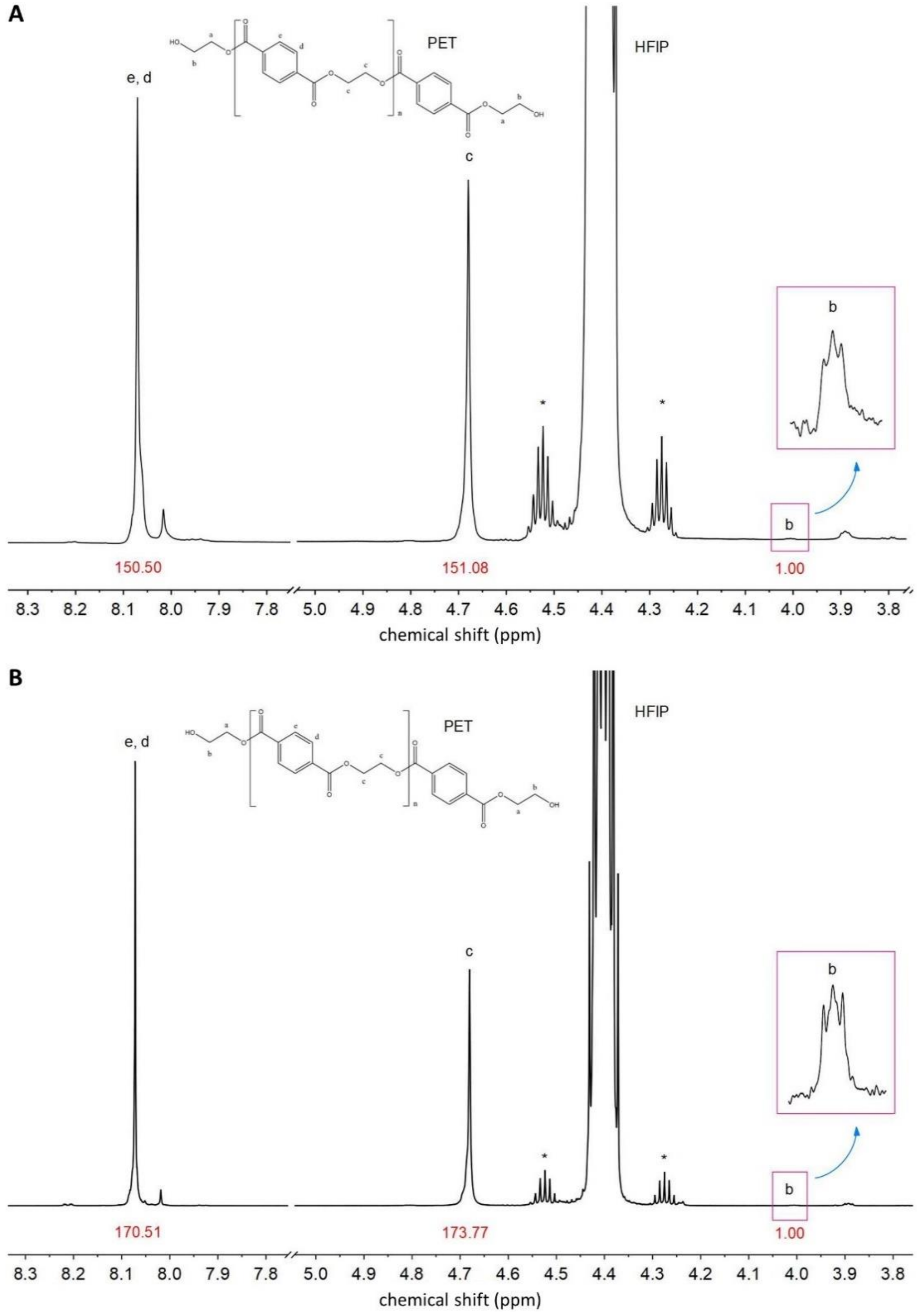

Figure S20: ^{1}H-NMR spectrum of PET synthesized with recovered TPA. (A) The two strong signals with δ^H of 8.07 and 4.69 ppm have been assigned to the protons in the aromatic ring (d, e) and the oxyethylene groups (c) in the repeating units. [11,15,16] An extraordinarily strong multiplet peak at 4.40 ppm originated from the solvent hexafluoroisopropanol (HFIP). The adjacent two multiple peaks are its ^{13}C satellite peaks.[11,16] A very weak signal was observed at 4.01 ppm related to the hydrogen (b) at the alpha position in the hydroxyl end groups. According to the ratio of the integration value of the peaks at 4.01, 4.69 and 8.07 ppm[11,16], an average DP of 151 was calculated, corresponding to an average molecular weight of 29,835 g mol^{-1}. (B) Reference ^{1}H-NMR spectrum of a G-PET film. An average DP of 172 was calculated, corresponding to an average molecular weight of 33,966 g mol^{-1}.

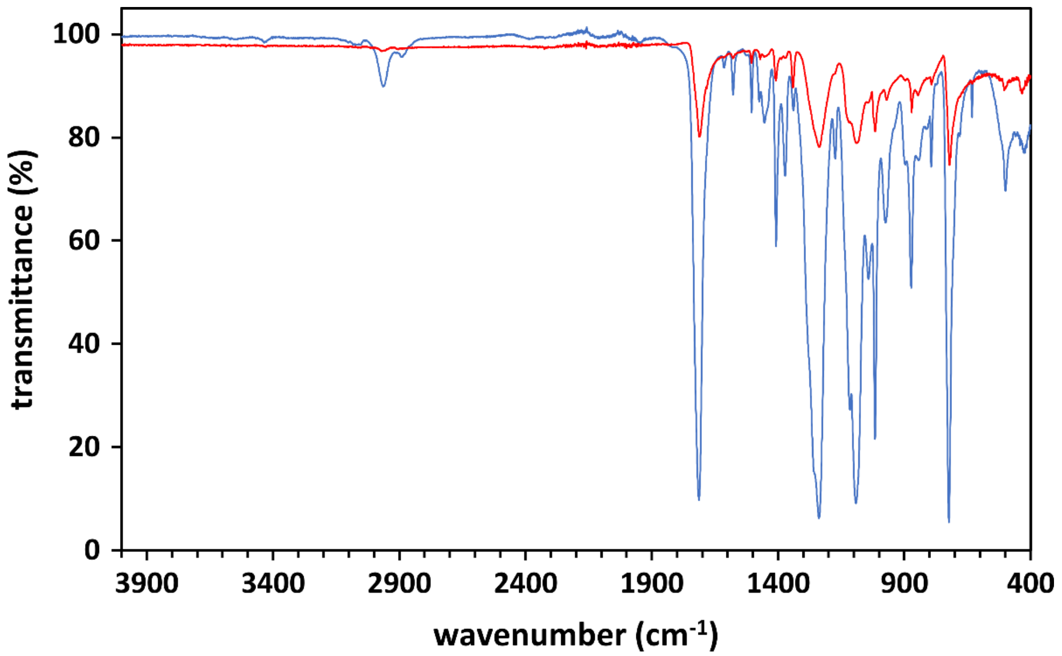

Figure S21: Fourier-transform infrared spectrum of synthesized PET (red). G-PET film was used as reference (blue).

References

[1] F. van den Ent, J. Löwe, *J. Biochem. Biophys. Methods* **2006**, *67*, 67–74.
[2] G. J. Palm, L. Reisky, D. Böttcher, H. Müller, E. A. P. Michels, M. C. Walczak, L. Berndt, M. S. Weiss, U. T. Bornscheuer, G. Weber, *Nat. Commun.* **2019**, *10*, 1717.
[3] W. Kabsch, *Acta Crystallogr. D Biol. Crystallogr.* **2010**, *66*, 125–132.
[4] P. R. Evans, G. N. Murshudov, *Acta Crystallogr. D Biol. Crystallogr.* **2013**, *69*, 1204–1214.
[5] A. J. McCoy, R. W. Grosse-Kunstleve, P. D. Adams, M. D. Winn, L. C. Storoni, R. J. Read, *J. Appl. Crystallogr.* **2007**, *40*, 658–674.
[6] A. Waterhouse et al., *Nucleic Acids Res.* **2018**, *46*, W296-W303.
[7] D. Ribitsch et al., *Biotechnol. Bioeng.* **2017**, *114*, 2481–2488.
[8] P. V. Afonine, R. W. Grosse-Kunstleve, N. Echols, J. J. Headd, N. W. Moriarty, M. Mustyakimov, T. C. Terwilliger, A. Urzhumtsev, P. H. Zwart, P. D. Adams, *Acta Crystallogr. D Biol. Crystallogr.* **2012**, *68*, 352–367.
[9] P. Emsley, K. Cowtan, *Acta Crystallogr. D Biol. Crystallogr.* **2004**, *60*, 2126–2132.
[10] E. F. Pettersen, T. D. Goddard, C. C. Huang, G. S. Couch, D. M. Greenblatt, E. C. Meng, T. E. Ferrin, *J. Comput. Chem.* **2004**, *25*, 1605–1612.
[11] F. Dieval, F. Khoffi, R. Mir, W. Chaouch, D. Le Nouen, N. Chakfe, B. Durand, *Int. J. Polym. Sci.* **2012**, *2012*, 1–14.
[12] P. Di Tommaso, S. Moretti, I. Xenarios, M. Orobitg, A. Montanyola, J.-M. Chang, J.-F. Taly, C. Notredame, *Nucleic Acids Res.* **2011**, *39*, W13-7.
[13] X. Han, W. Liu, J.-W. Huang, J. Ma, Y. Zheng, T.-P. Ko, L. Xu, Y.-S. Cheng, C.-C. Chen, R.-T. Guo, *Nat. Commun.* **2017**, *8*, 2106.
[14] a) E. Ghamary, M. M. A. Nikje, S. L. R. Andabil, L. Sarchami, *Polímeros* **2018**, *28*, 1–5; b) F. Quartinello, S. Vajnhandl, J. Volmajer Valh, T. J. Farmer, B. Vončina, A. Lobnik, E. Herrero Acero, A. Pellis, G. M. Guebitz, *Microb. Biotechnol.* **2017**, *10*, 1376–1383;
[15] Z. Han et al., *Materials* **2018**, *11*.
[16] P. Falkenstein, D. Gräsing, P. Bielytskyi, W. Zimmermann, J. Matysik, R. Wei, C. Song, *Front. Microbiol.* **2020**, *11*, 689.
